# Revealing nuclear receptor hub modules from Basal-like breast cancer expression networks

**DOI:** 10.1371/journal.pone.0252901

**Published:** 2021-06-23

**Authors:** Sharon Nienyun Hsu, Erika Wong En Hui, Mengzhen Liu, Di Wu, Thomas A. Hughes, James Smith

**Affiliations:** 1 School of Food Science & Nutrition, Faculty of Environment, University of Leeds, Leeds, United Kingdom; 2 Astbury Centre, Faculty of Biological Sciences, University of Leeds, Leeds, United Kingdom; 3 School of Medicine, University of Leeds, Leeds, United Kingdom; Centro Nacional de Investigaciones Oncologicas, SPAIN

## Abstract

Nuclear receptors are a class of transcriptional factors. Together with their co-regulators, they regulate development, homeostasis, and metabolism in a ligand-dependent manner. Their ability to respond to environmental stimuli rapidly makes them versatile cellular components. Their coordinated activities regulate essential pathways in normal physiology and in disease. Due to their complexity, the challenge remains in understanding their direct associations in cancer development. Basal-like breast cancer is an aggressive form of breast cancer that often lacks ER, PR and Her2. The absence of these receptors limits the treatment for patients to the non-selective cytotoxic and cytostatic drugs. To identify potential drug targets it is essential to identify the most important nuclear receptor association network motifs in Basal-like subtype progression. This research aimed to reveal the transcriptional network patterns, in the hope to capture the underlying molecular state driving Basal-like oncogenesis. In this work, we illustrate a multidisciplinary approach of integrating an unsupervised machine learning clustering method with network modelling to reveal unique transcriptional patterns (network motifs) underlying Basal-like breast cancer. The unsupervised clustering method provides a natural stratification of breast cancer patients, revealing the underlying heterogeneity in Basal-like. Identification of gene correlation networks (GCNs) from Basal-like patients in both the TCGA and METABRIC databases revealed three critical transcriptional regulatory constellations that are enriched in Basal-like. These represent critical NR components implicated in Basal-like breast cancer transcription. This approach is easily adaptable and applicable to reveal critical signalling relationships in other diseases.

## Introduction

Nuclear receptors (NRs) are ligand induced transcriptional factors that regulate essential pathways in normal physiology and in disease. With the advantage of next generation sequencing technology, researchers are beginning to comprehend the innate complexity of signalling pathways involving NRs and their involvement in tumour development and progression [1]. In this work, we used an unsupervised clustering method to stratify breast cancer into classes with distinct NR expression patterns and identified three critical modules that are enriched in Basal correlation networks. Network-based modules provide a robust description of transcriptional characteristics underlying Basal-like breast cancer, and also take into account dynamic properties and collaborative behaviours of NRs.

### Basal-like breast cancer

Breast cancer is not a single disease and consists of a high degree of genomic heterogeneity [2]. Consequently, diversification of therapeutic treatments is required for all breast cancer patients. Previous work has disambiguated the heterogeneity, Perou *et al.* and Sørlie *et al.* revealed five molecular subtypes (Luminal A, Luminal B, ERBB2(Her2), Basal-like and normal-like) with each representing a distinct molecular portrait and prognostic outcome [3–6]. Debate continues as to whether normal-like subtype represent cancerous tissue. Identification of the other four molecular subtypes provides useful insight into understanding the tumour development and helps to tailor clinical decisions [7, 8]. Luminal A, Luminal B and Her2 subtype are defined by the presence of oestrogen receptor (ER), progesterone receptor (PGR) and the human epidermal growth factor receptor 2 (HER2) and respond to selective treatments that target these markers [9]. Tumours lacking these three receptors are referred to as triple negative breast cancer (TNBC) and such patients receive conventional cytostatic and cytotoxic chemotherapy that is non-selective typically systemic. Moreover, most tumours with TNBC status are classified as Basal-like subtype based on PAM50 subtype prediction scheme [10, 11]. Basal-like subtype has a high expression of cytokeratin (CK 5/6, CK14, and CK17) and genes involved in cell proliferation. Compared to other breast cancer subtypes, Basal-like is particularly aggressive and patients are at high risk for relapse and death within the first 2 to 5 years of diagnosis [12, 13]. Among all the molecular subtypes, Basal-like remains a greatest clinical challenge due to its aggressive nature and poorly-characterised molecular pathogenesis. The devastating clinical outcome and limited treatment options have motivated this research. The aim of this work is reveal the unique transcriptional characteristics underlying Basal-like subtype.

### The significance of nuclear receptor in breast cancer

Nuclear receptors (NRs) are a superfamily of ligand-induced transcription factors, regulating both physiological and pathological processes. Previous studies have demonstrated the involvement of individual NRs in breast cancer initiation and progression [14, 15].

NR expression data can also be interpreted together to provide a valuable estimation on breast cancer recurrence and prognosis [16]. It has been demonstrated that NRs have distinct expression levels in different breast cancer subtypes, suggesting that the transcriptional activity underlying different breast cancer subtypes are distinct and require different therapeutic strategies [17–19].

NR co-regulators interact with both NRs and other transcription factors to facilitate transcription of target genes. They are essential for recruiting protein complexes that are involved in NR-regulated signalling pathways. As transcriptional events are specific and tightly regulated, an aberrant alteration (over or under-expression) of NRs or their co-regulators can lead to pathogenesis [20]. Early work established that the expression level of NR co-regulators modulate tumour cell responsiveness to hormonal therapy in breast cancer [16]. It is clear that understanding the functions and transcriptional dynamics of NR co-regulators will improve treatment response prediction and open up more opportunities for targeting oncogenic pathways modulated by NRs.

### Biological networks

Understanding the genetic diversities underlying breast cancer tumours is crucial for targeted treatment development and it should be recognised that biological systems are complex, and phenotypic diversity is indeed a reflection of a combination of subtle dynamic interactions rather than individual genetic alterations (eg mutations, transcriptions, regulation events).

The development of next generation sequencing (NGS) has provided rapid identification of genetic variations implicated in cancer. However, initial studies often focussed on identifying differential expression or molecular alterations of individual biomolecules (e.g. DNA, mRNA or protein) and less so on the dynamic properties and collaborative behaviours in the biological complexity at the system level [21, 22]. Therefore, to develop and improve the efficiency of cancer-targeted treatment, more effort is required to model the cellular transcription signalling dynamics and capture the underlying molecular state driving oncogenesis [23, 24].

Network-based approached have emerged as powerful approaches for interpreting biological data, including expression data (e.g. genome, transcriptome). They can provide a system-level understanding of the biological mechanisms and constituent relationships in diseases including breast cancer [25–27].

In this work, gene correlation networks are described for Basal-like breast cancer, from two independent patient cohort databases of invasive breast carcinoma: TCGA (www.cbioportal.orgstudysummary?id=brca_tcga_pub) [28] and METABRIC (www.cbioportal.orgstudysummary?id=brca_metabric) [29]. From these networks, we identified hub-associated modules that are enriched and conserved across two independent breast cancer cohorts. These modules contain critical NRs that are operating in functional units, annotated as small network motifs. Graphically, these hub-associated NR motifs are topologically central with a maximal number of connections, representing critical positions in the network for targeting. Biologically, these motifs are network-based indicators of essential components underlying Basal-like transcriptional regulatory signalling, providing a mechanistic understanding of Basal-like breast cancer characteristics.

## Materials and methods

In this work, mRNA gene expression data from individual patients were clustered using a computational pipeline written in R. Cluster analysis required R (v3.5.3), R Studio(v1.2.5019), R/Bioconductor (3.10), R/BHC (v1.38.0) [30, 31]. Code used for the clustering is provided in S3.6 File in S1 Appendix, for reproducibility. Network modelling required R/GeneNet (1.2.13) [32–34], and R/Graphiz (v2.26.0). Terminology and abbreviations used in the paper are described in S1 Appendix.

### Overview of the unsupervised clustering analysis protocol

**Fig 1:**
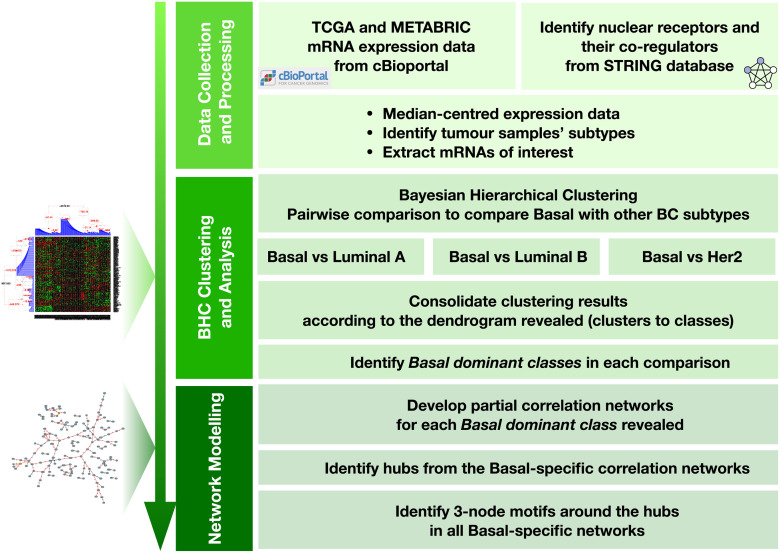
Overview of our approach for revealing critical modules in Basal-specific networks. Our method has three main steps, including (a) data collection and preparation, (b) breast cancer stratification from unsupervised clustering and (c) network modelling and analysis. Pictures on the left show a typical outcome from each step.

The unsupervised clustering method used here revealed a natural stratification of breast cancers from two large independent breast cancer patient cohorts. By combining an unsupervised clustering method with network modelling, we identified critical 3-node motifs that are enriched in Basal-like. These network motifs represent critical modules in the Basal-specific networks, providing fundamental knowledge for developing more targeted therapeutics for Basal-like patients. The overview of our strategy is shown in Fig 1.

### TCGA expression data

TCGA (The Cancer Genome Atlas Program) breast tumour mRNA gene expression data from 825 patients was accessed from cBioportal, [28]. Three hundred and eleven tumour samples were not assigned to any PAM50 subtype were therefore eliminated from the analyses. The remaining samples totalled 514 breast tumour samples, consisting of 231 Luminal A samples, 127 Luminal B samples, 58 Her2 samples and 98 Basal-like samples (Table 1). The subtype of each tumour sample was based on the PAM50 molecular subtypes proposed by Parker [10]. The mRNA expression data from the TCGA study is organised into levels and level 3 was used in this study. Level 3 includes the least processed. The expression signals were median-centred to account for the potentially skewered distributions of the differentially expressed genes. Out of the 178 NR-associated genes, 171 were available in TCGA and included in the TCGA analyses and their mRNA expression are referred to as NR-associated gene signals throughout this paper. The 178 genes contain 46 NR genes and 132 genes that code for their binary interacting partners (NR co-regulators). The gene list is described in S2 Appendix) and the known NR co-regulators were described and annotated in the STRING database (v11.0 https://string-db.org/).

**Table 1:** Patients subtype distribution in TCGA and METABRIC. Subtypes considered in this study are highlighted in bold.

	Luminal A	Luminal B	Her2	Basal-like
TCGA	231	127	58	98
METABRIC	679	461	220	199

### METABRIC expression data

The METABRIC (Molecular Taxonomy of Breast Cancer International Consortium) mRNA gene expression data from 1565 patients was also obtained from cBioportal (*data_expression.txt* from the compressed file) [29]. Six patients were eliminated from the analysis due to insufficient clinical information, leaving a total number of 1559 breast tumour samples. This included 679 Luminal A samples, 461 Luminal B samples, 220 Her2 samples and 199 Basal-like samples (Table 1). The subtype of each tumour was based on PAM50 molecular subtypes and the assignment can be found from the file: *data_clinical_supp_patient.txt* in the publicly available compressed file: *brca_METABRIC.tar.gz*. To make the analyses compatible with TCGA, patients with claudin-low and normal-like subtypes were not considered in the analyses. Unlike the TCGA data, these expression signals were median-centred in R, to account for the potentially skewered distributions of the differentially expressed genes. Out of the 178 NR-associated TCGA genes, from above, 169 were available in METABRIC and included in the METABRIC analyses and their mRNA expression are referred to as NR-associated gene signals throughout this paper. As above, the 178 genes contain 46 NR genes and 132 genes that code for their binary interacting partners (NR co-regulators) (see S2 Appendix) and the known NR co-regulators were described and annotated in the STRING database (v11.0 https://string-db.org/).

### Unsupervised Bayesian Hierarchical Clustering

In this work, patients were grouped into clusters by Bayesian Hierarchical Clustering (BHC) based on their transcriptome variation [35, 36]. To simplify the patient groupings, the resulting clusters were further enumerated into classes, defined according to the final or penultimate fusions in the dendrogram. The original clusters revealed and the clusters-to-classes simplification process are provided and illustrated in S3.2 File in S3 Appendix. Importantly, the choice of classes still produces clear groups. However indications of uncertainty, compared to a null model from a background population giving a probability for statistical significance, are not available from R/BHC.

Groups of patients in the classes reflect the distribution of the patients in the clinical subtypes. Depending on the patients present within a class, classes were defined as either a *subtype dominant class* or an *ambiguous class*. *Subtype dominant classes* are said to be homogeneous, containing patients predominantly of one subtype. *Ambiguous classes* are heterogenous containing a similar number of patients from more than one subtype. For the purpose of this work, only *Basal dominant classes* were considered and analysed. Overlaps between different *Basal dominant classes* are illustrated in S3.4 and S3.5 Files in S3 Appendix.

BHC can be performed in two-dimensions giving rise to alternative Cluster Analysis Strategies, with one using the transform of the data. This work only considers the patients clustered according to the distributions of mRNA gene expression. A complementary analysis uses the transform of the data, resulting in clustering mRNA gene expression. The two clustering strategies are explained in S3.4 File in S3 Appendix. The analyses including both patients clustered by genes and genes clustered by patients are provided in S3.4 and S3.5 Files in S3 Appendix.

### Partial correlation networks

To identify critical NRs and their associated functional units in Basal-like, the partial correlation networks were derived from the Basal-like dominant classes using the R/GeneNet library [32]. The function R/GeneNet:network.test.edges was used to create a pairwise partial correlation matrix containing all possible correlations, listed by order of magnitude. Marginalised thresholding of 1% was used to reveal the stronger correlations and to eliminate the weaker correlations. R/Graphiz was used to generate networks for visualisation. The partial correlation networks presented in this work are signed (containing positive and negative correlations) and undirected. Each node in the network represents a gene and the lines between them represent a strong (top 1%) correlation. Each node was marked with its degree, defined as the number of connections that a given node makes in its network. The node degrees were obtained from GeneNet::node.degree and the total degree being the sum of degrees each node makes across the eight Basal-specific networks. All nodes were then ranked by their total degree. The total degree distribution of the nodes was also plotted for hub identification. Hubs are the top three nodes with the highest degree. Code used for network analysis were adapted from this GeneNet example (http://www.strimmerlab.org/software/genenet/download/ecoli-net.R).

### Hub-associated local networks and 3-node motifs

Once hubs are identified, the hub-associated local network can be defined by 3-node network motifs. Hub-associated local networks are defined by the inclusion of all possible 3-node motifs within the radial proximity of two edges from a given hub node. In this work, 3-node motifs centred around the top two most connected nodes were annotated and compared for all networks derived from *Basal dominant classes* (see S5 Appendix). Motifs and hub-associated local networks were curated manually and systematically checked to eliminate human error in the annotation process.

## Results

This work employed a nonparametric multinomial *Dirichlet* process clustering algorithm, Bayesian Hierarchical Clustering (BHC) to stratify breast cancer patients and discriminate the Basal-like patients based on the mRNA expression signals of nuclear receptors and their immediate co-regulators. In order to reveal characteristics that are unique to Basal-like, pairwise comparisons were performed. Individually, Basal-like was compared with Luminal A, and with Luminal B and with Her2 subtypes. The aim of this study was to uncover unique transcriptional regulatory constellations that suggest dynamic relationships between critical NRs and their co-regulators underlying Basal-like breast cancer only, given the data from the two independent datasets, TCGA [28] and METABRIC [29].

### Breast cancer stratification and pairwise subtype comparison

When two subtypes are compared, patients are expected to fall into either a *subtype dominant class* or an *ambiguous class*. In a *subtype dominant class* the majority of patients will have the same PAM50 subtype and in an *ambiguous class* patients have a mixture of both subtypes. The patients in the *subtype dominant class* were expected to have distinct and relatively simple characteristics whereas the patients in the *ambiguous classes* were considered to have more complex transcriptional activities. This work focuses on revealing distinct characteristics of patients found in the most homogenous *Basal dominant classes*.

Our script with BHC produced colour plots (modified heatmaps) with hierarchies (dendrograms) on both axes revealing the latent structure the data. The clustering produces a more natural stratification of the patients, according to the NR expression patterns. In the following analyses, a corresponding 2D colour plot aligned with the dendrograms giving the hierarchy of the patient clusters. Each gene is a considered as a continuous variable and its distribution was discretised into three (an upper-bound indicating the highest expression, the marginal-likelihood representing the signal, and a lower-bound indicating the lowest expression) prior to clustering.

#### Basal-like vs Luminal A

**Fig 2:**
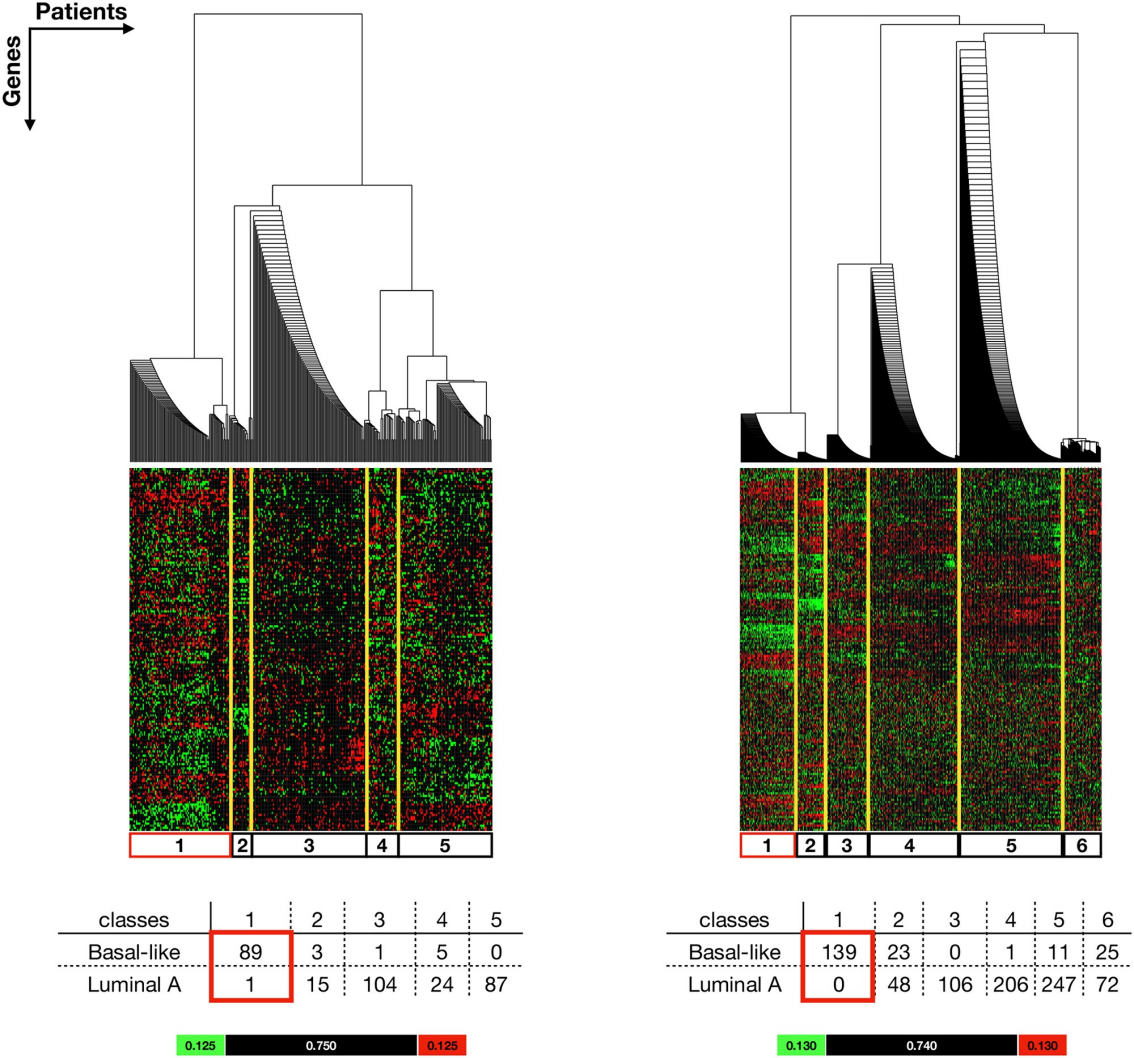
Extracted results from Basal-like vs Luminal A comparison show a natural stratification of Basal-like vs Luminal A patients (columns) according to the NR-associated gene expression signals (rows). See S3.4 and S3.5 Files in S3 Appendix for the complete results. **Left:** Patients from TCGA dataset were grouped into 5 classes. Class 1 is considered as a *Basal dominant class*. **Right:** Patients from METABRIC dataset were grouped into 6 classes. Class 1 is considered as a *Basal dominant class*. Yellow lines highlight the classes (simplified from the clusters) and the corresponding expression pattern in the colour plot. The tables show the number of Basal-like and Luminal A patients in each class. The *Basal dominant classes* are highlighted in red boxes in the table and colour plots. The colour bar indicates the lowerbound (green), likelihood (black) and upperbound (red).

In the Basal-like vs Luminal A analysis, 329 patients (98 Basal-like and 231 Luminal A) from TCGA database were grouped into 5 classes (Fig 2, **Left**). Similarly, 878 patients (199 Basal-like and 679 Luminal A) from METABRIC were grouped into 6 classes (Fig 2, **Right**). The classes are simplifications of the clusters revealed by BHC, representing patient groups with distinct NR expression patterns. In both analyses, class 1 are considered as *Basal dominant classes*, as they consist mostly Basal-like patients with only a single Luminal A patient in TCGA class 1. Class 3, 4 and 5 from both the TCGA cluster analysis and the METABRIC cluster analysis are considered as *Luminal A dominant classes* as the majority of the patients in these classes are Luminal A patients. TCGA Class 2 and METABRIC classes 2 and 6 are considered as *ambiguous classes* as they consist a similar number of Basal-like and Luminal A patients within a class. The hierarchy (in the top dendrograms) shows that *Basal dominant classes* from both analyses are separated from the *Luminal A dominant classes* at the top divisions, indicating that the patients in *Basal dominant classes* have very distinct nuclear receptor expression patterns compared to those in the Luminal A dominant classes.

#### Basal-like vs Luminal B

**Fig 3:**
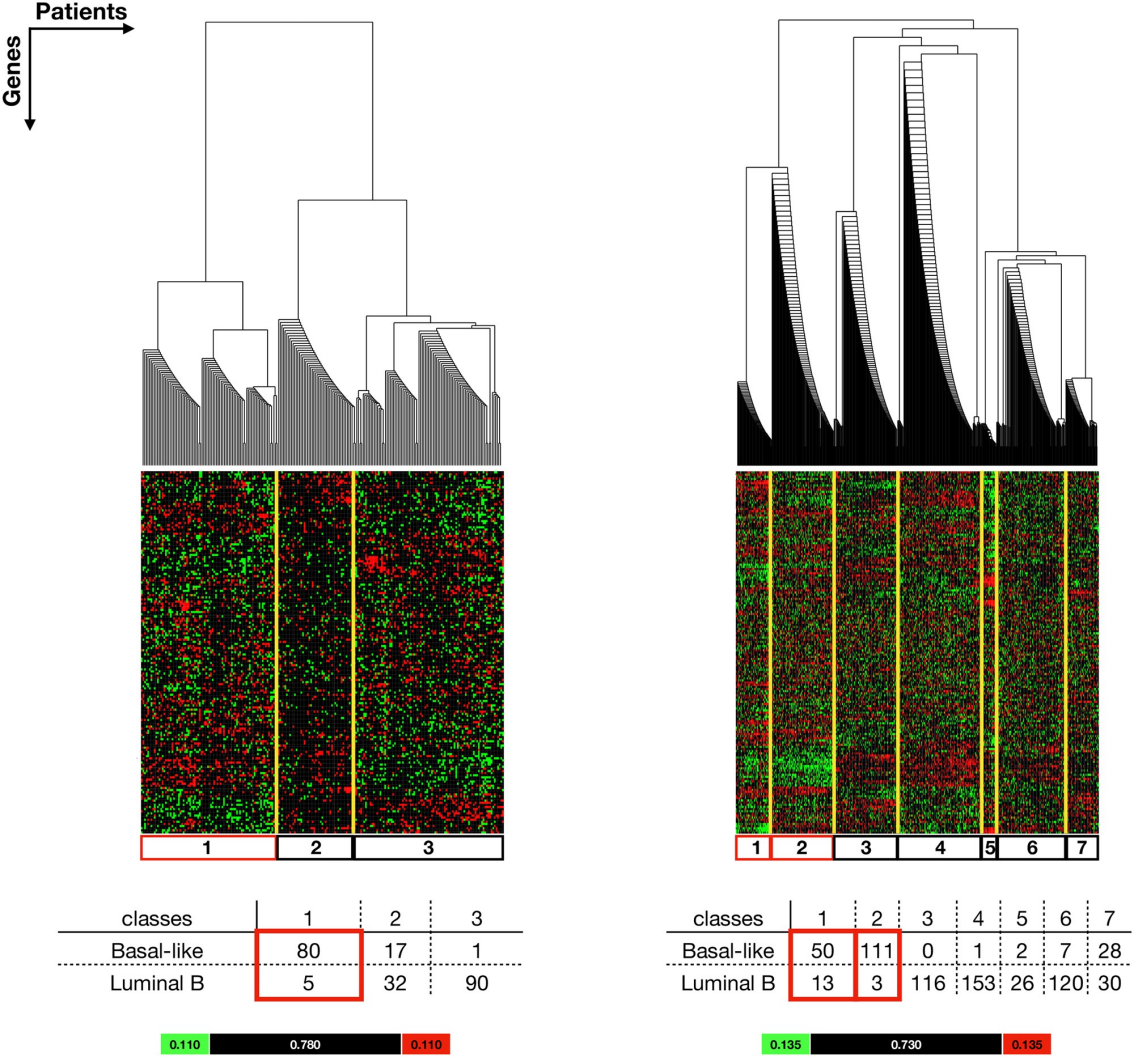
Extracted results from Basal-like vs Luminal B comparison show a natural stratification of Basal-like vs Luminal B patients (columns) according to the NR-associated gene expression signals (rows). See S3.4 and S3.5 Files in S3 Appendix for the complete results. **Left:** Patients from TCGA dataset were grouped into 3 classes. Class 1 is considered as a *Basal dominant class*. **Right:** Patients from METABRIC dataset were grouped into 7 classes in which class 1 and class 2 are considered as *Basal dominant classes*. Yellow lines highlight the classes (simplified from the clusters) and the corresponding expression pattern in the colour plots. The tables show the number of Basal-like and Luminal B patients in each class. The *Basal dominant classes* are highlighted in red boxes in the table and colour plots. The colour bar indicates the lowerbound (green), likelihood (black) and upperbound (red).

Similar results were obtained in the Basal-like vs Luminal B comparisons. Patients were clustered into 3 classes from the TCGA analysis and 7 classes from METABRIC analysis (Fig 3). TCGA class 1 and METABRIC classes 1 and 2 were considered as *Basal dominant classes*. However, METABRIC class 1 is more heterogeneous as it contains 50 Basal-like patients and 13 Luminal B patients compared to class 2 that contains 111 Basal-like patients and only 3 Luminal B patients. Consequently, we therefore considered METABRIC class 1 to be an insubstantial *Basal dominant class*. In both TCGA and METABRIC analyses, there is discrimination at the top division of the hierarchy between the *Basal dominant classes* from *Luminal B dominant classes* and the *ambiguous classes*. This indicates that in general, Basal-like and Luminal B patients have very distinct nuclear receptor expression signals.

#### Basal-like vs Her2

**Fig 4:**
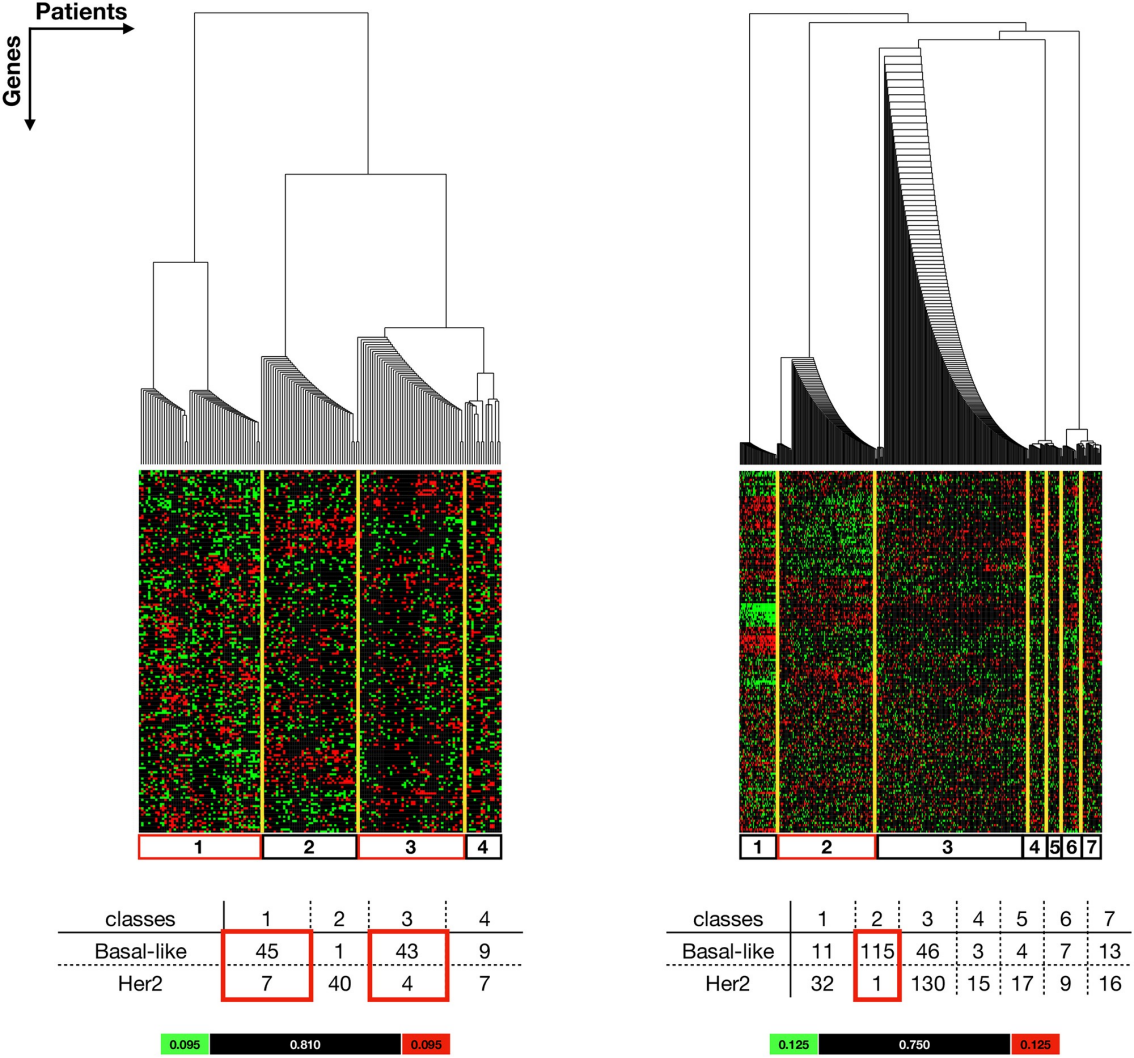
Extracted results from Basal-like vs Her2 comparison show a stratification of Basal-like vs Her2 patients (columns) according to the NR-associated gene expression signals (rows). See S3.4 and S3.5 Files in S3 Appendix for the complete results. **Left:** Patients from TCGA dataset were grouped into 4 classes. Class 1 and class 3 are considered as *Basal dominant classes*. **Right:** Patients from METABRIC dataset were grouped into 7 classes. Class 2 is considered as a *Basal dominant class*. Yellow lines highlight the classes (simplified from the clusters) and the corresponding expression pattern in the colour plot. The tables show the number of Basal-like and Her2 patients in each class. The *Basal dominant classes* are highlighted in red boxes in the table and colour plots. The colour bar indicates the lowerbound (green), likelihood (black) and upperbound (red).

In the Basal-like vs Her2 comparisons, some Basal-like patients are distinguishable from Her2 patients. However, the separation of Basal-like and Her2 are not as distinct as the Basal-like vs Luminal type comparisons. In the TCGA analysis (Fig 4 **Left**), patients clustered into 4 classes and two of which were *Basal dominant classes* (class 1 and class 3). Interestingly, the two *Basal dominant classes* emerge from different descenders at the top division of the hierarchy, suggesting distinct characteristics. Class 1 is from the left descender and is separated from the rest of the classes, whereas class 3 is from the right descender with the *Her2 dominant class* (class 2) and the *ambiguous class* (class 4). TCGA class 3 shares more similarity with Her2 therefore is considered as an insubstantial *Basal dominant class*. In the METABRIC analysis (Fig 4 **Right**), patients clustered into 7 classes. More than half of the Basal-like patients (115 out of 199 Basal-like patients) appear in *Basal dominant class* (class 2), the remainder of Basal-like patients were distributed across the other six classes. In particular, 46 out of 199 Basal-like patients were grouped into the *Her2 dominant class*, suggesting that these patients have similar nuclear receptor expression characteristics to Her2 patients. The clustering shows clearly that while most Basal-like patients have distinct NR expression patterns from Luminal type patients, a fraction of Basal-like patients have more similar NR expression patterns to Her2 compared to Basal-like. This observation is consistent in both the TCGA and METABRIC datasets used in this work.

### Basal-specific nuclear receptor networks

**Fig 5:**
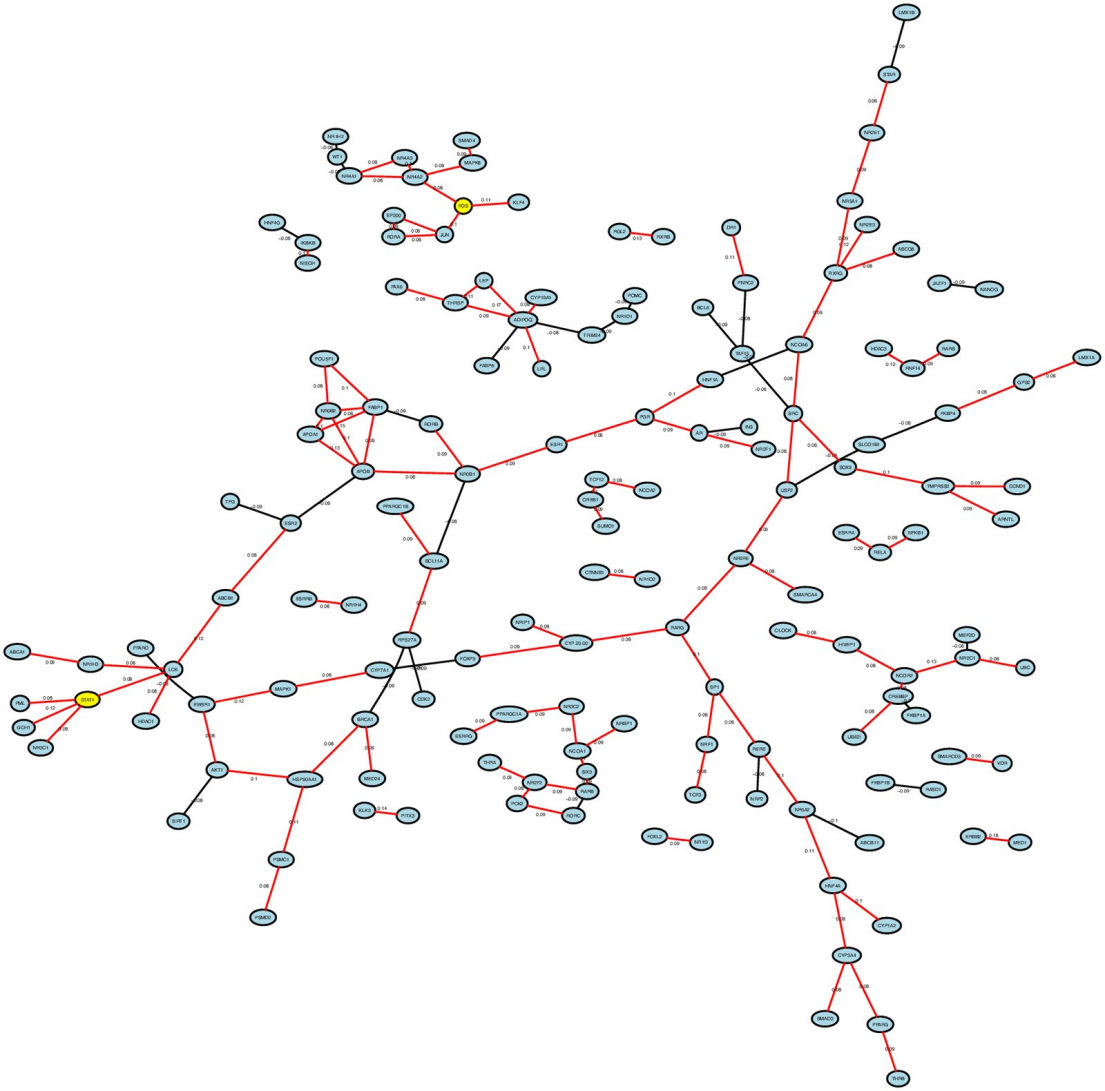
An example of Basal-specific partial correlation networks constructed from *Basal dominant class* TCGA class 1 from the Basal-like vs Luminal A comparison. Each node represents a gene and the lines between the nodes represent a correlation. Red lines represent positive correlations and black lines represent negative correlations. The numbers next to the lines represent the strength of the partial correlations. Node FOS, STAT1 are the most connected nodes across the Basal-specific networks, highlighted in yellow.

**Fig 6:**
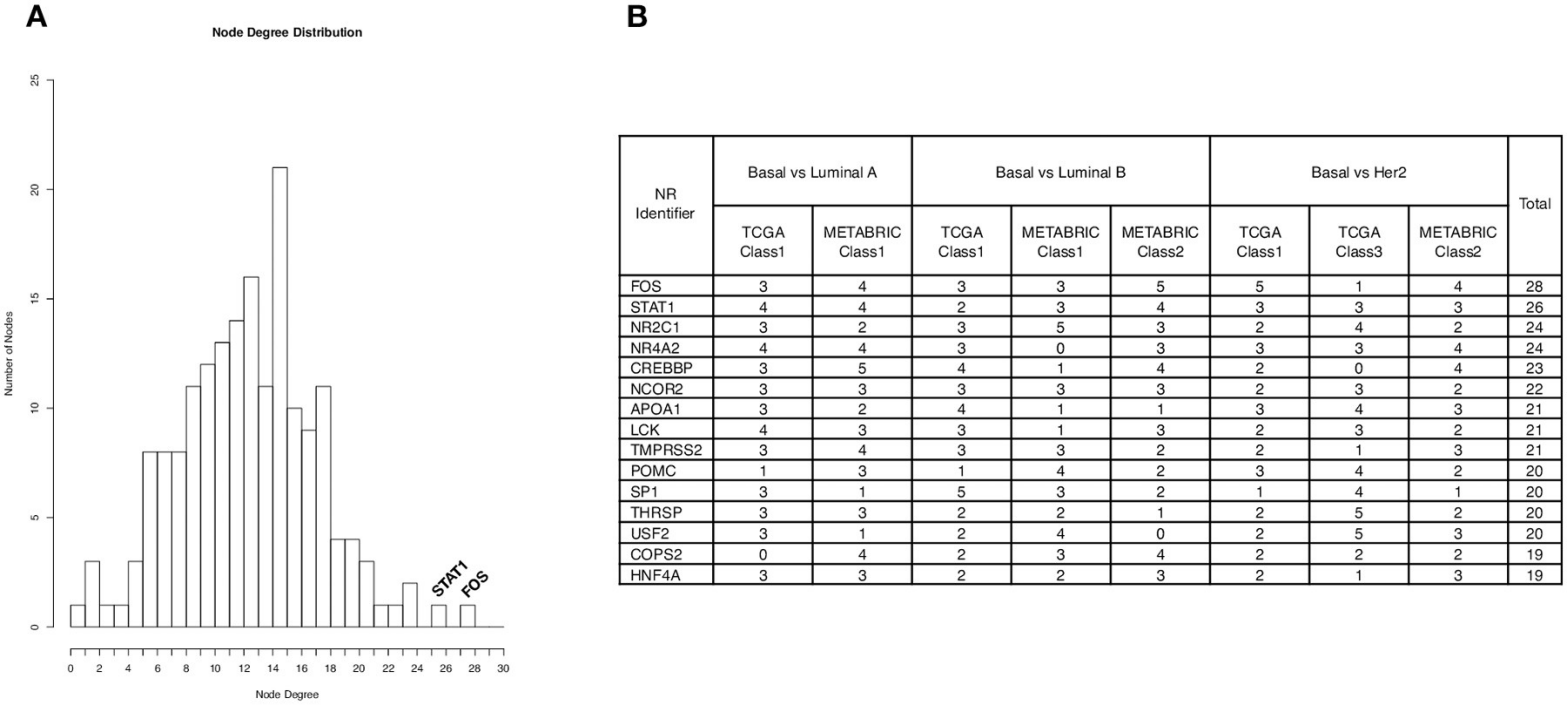
Node degree summary. **A:** The histogram shows the distribution of total degree of the nodes. FOS and STAT1 are the most connected nodes across networks with total degree of 28 and 26, respectively. They are defined as hubs and co-expression around the hubs was explored. **B:** The 15 most connected genes listed by the total number of connections in all Basal-specific networks.

To reveal the distinct characteristic for Basal-like patients, we developed partial correlation networks for each of the *Basal dominant classes* revealed from the analysis. The networks were developed based on the available NR-associated genes and only the strongest 1% of the correlations were included in the networks. The networks are undirected and signed, containing positive and negative correlations between the nodes (genes). The positive correlations are shown in red and the negative correlations are shown in black in the networks. Fig 5 shows an example of a partial correlation network, developed for the *Basal dominant class* from the TCGA Basal-like vs Luminal A comparison. Partial correlation networks derived from other *Basal dominant classes* can be found in S4 Appendix. To identify the critical genes across networks, a frequency analysis was performed to summarise the degree of connectivity of a given node in all eight Basal-specific networks. All nodes are ranked according to the magnitude of total degree (connection) across networks. Fig 6A shows the distribution of total degree of the nodes and the table in Fig 6B shows the top 15 most connected nodes. The complete summary table containing all 178 nodes and their corresponding degrees across networks is shown in S6 Appendix. FOS and STAT1 are the most connected nodes across the networks and are defined as hubs. Hub genes occupy central positions in the network, representing a stronger capacity to modulate adjacent genes than the genes with the lower degrees. Therefore, correlations around FOS and STAT1 were explored further in this work.

### Critical 3-node motifs

In order to reveal critical modules from Basal-specific networks, the hub nodes (FOS and STAT1) were examined further. From the partial correlations hub-associated undirected 3-node motifs were identified that were self-consistent within and between the analyses. Three-node network motifs can be considered as the smallest functional units, often used as indicators for revealing functional properties in complex transcription networks.

**Fig 7:**
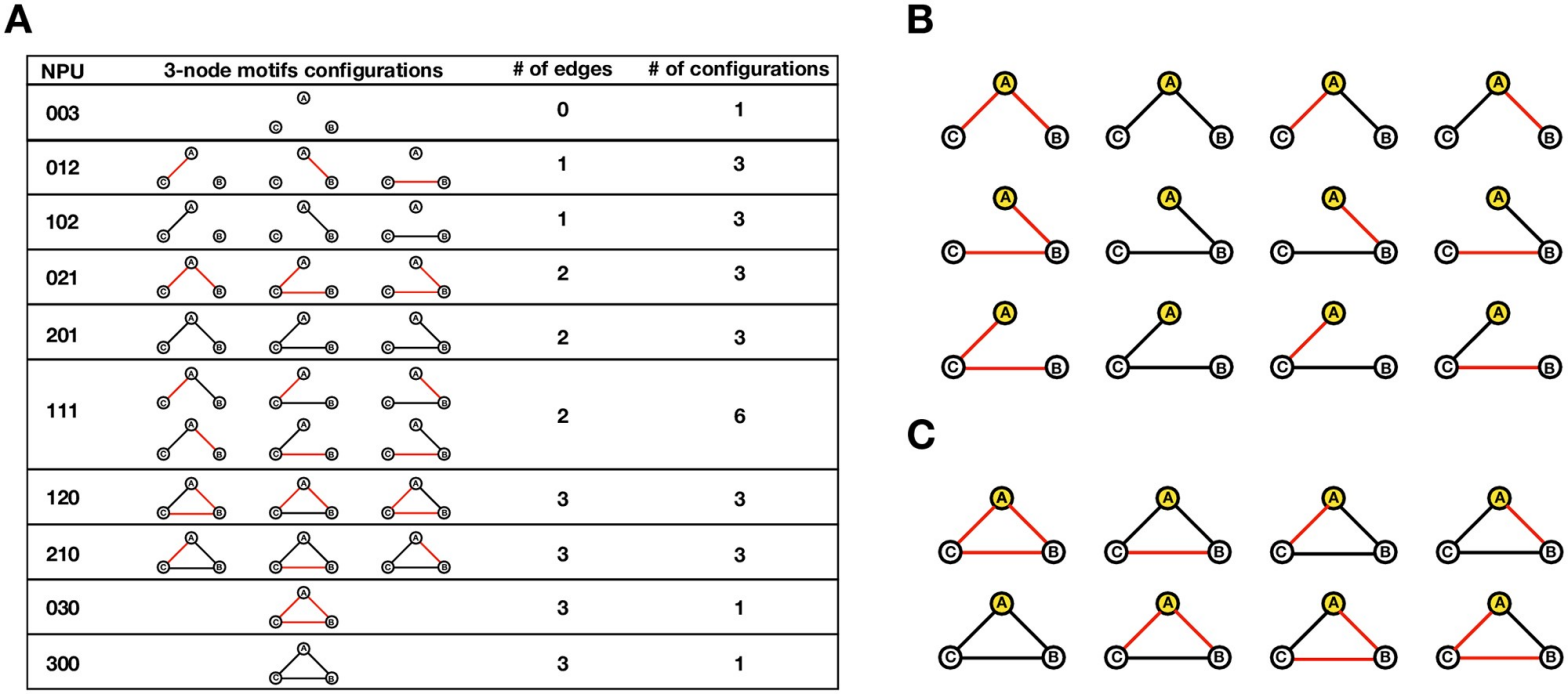
Classification of 3-node motifs adopted from the *triad census* proposed by Davis and Leinhardt [37]. Nodes are labelled A, B, C clockwise to represent non-redundant NRs. Edges are illustrated as positive correlations (stronger than +1.0%) in red or as negative correlations (stronger than -1.0%) in black. **(A)** The 27 NPU configurations of non-redundant nodes. **(B)** The 12 configurations of the linear motifs based on the position of the hub (labelled A and in yellow). Top row: node A is connected to both B and C. Middle row: node A is connected to C via B. Bottom row: node A is connected to B via C. **(C)** The 8 configurations of the non-redundant complete 3-node network motifs. These motifs are divided into two groups based on the position of the hub (labelled A and in yellow). Top row: Node A and associated nodes B and C and are considered to have coherent behaviour. Bottom row: Node A and associated nodes B and C and are considered to have incoherent behaviour.

Fig 7A lists the configurations of 3-node motifs from the signed correlation networks. We adopted a *triad census*, proposed by Davis and Leinhardt [37] to classify the 27 3-node motifs into 10 groups, where each group is given a unique NPU (Negative, Positive, Un-associated) code [38, 39]. The first number represents the number of Negative correlations (black), the second number is the number of Positive correlations (red) and the third number represents the number of Un-associated correlations in a given 3-node motif. NPU code 003 represent three un-associated nodes seemingly independent. In this work, the correlations in the motifs represent paired associations between three nodes that are stronger than the 1.0% threshold to justify being included as edges. The 3-node motifs can have isomorphs for example NPU code 111 has six variants. NPU codes 021, 201 and 111 contain 3 nodes and 2 edges, representing 12 linear topologies (Fig 7B). NPU codes 120, 210, 030 and 300 all contain 3 nodes and 3 edges, representing 8 complete topologies (Fig 7C).

**Fig 8:**
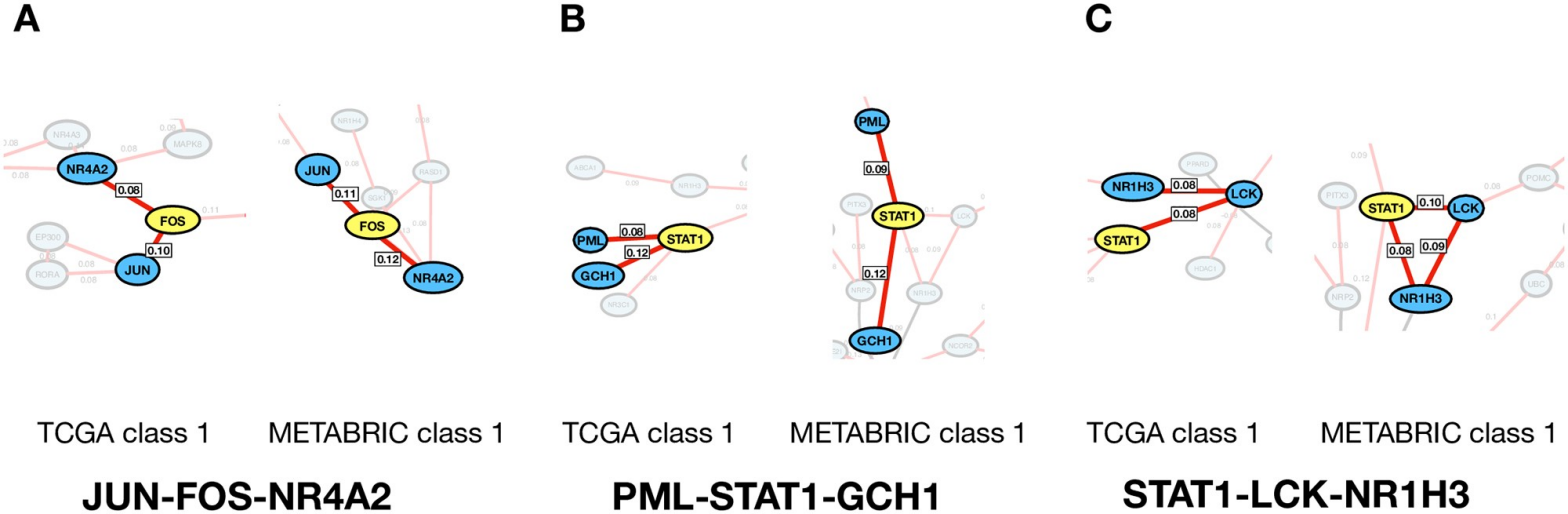
Three critical 3-node motifs embedded in the cropped Basal-specific networks from the Basal-like vs Luminal A comparison (TCGA and METABRIC). The 3-node network motifs are highlighted in bold in each network and are observed in the other four Basal-specific networks. See S7 Appendix for details of the networks from the other comparisons. **(A)** Motif JUN-FOS-NR4A2. **(B)** Motif PML-STAT1-GCH1. **(C)** Motif STAT1-LCK-NR1H3.

The hub-associated local networks are defined by 3-node motifs around the hubs, including two levels of edges from the hub (see S5 Appendix). Table 2 summarises the occurrence of all the linear (021, 201, 111) and complete (120, 210, 030, 300) motifs centred around FOS and STAT1 in each of the Basal-specific networks. In most networks, type 021 is the most common type of 3-node motif followed by type 111 and then type 030. Three 3-node motifs JUN-FOS-NR4A2, PML-STAT1-GCH1 and STAT1-LCK-NR1H3 were found to be enriched in the Basal-specific networks from both the TCGA and METABRIC datasets (Fig 8, Table 3).

**Table 2:** Occurrence of the 7 types of NPU motifs centred around FOS and STAT1 from Basal-specific networks. Numbers in the table indicate the frequency of each NPU motif occurring in the corresponding hub-associated local networks.

FOS		**021**	**201**	**111**	**120**	**210**	**030**	**300**
Basal-like vs Luminal A	TCGA Class1	8	0	0	0	0	2	0
	METABRIC Class 1	8	0	0	0	0	2	0
Basal-like vs Luminal B	TCGA Class1	7	0	1	0	0	0	0
	METABRIC Class 1	4	0	2	0	0	0	0
	METABRIC Class 2	8	0	6	0	0	1	0
Basal-like vs Her2	TCGA Class1	14	0	2	0	0	0	0
	TCGA Class3	0	0	0	0	0	0	0
	METABRIC Class 2	9	0	1	0	0	2	0
STAT1		**021**	**201**	**111**	**120**	**210**	**030**	**300**
Basal-like vs Luminal A	TCGA Class1	9	0	0	0	0	0	0
	METABRIC Class 1	8	0	1	0	0	1	0
Basal-like vs Luminal B	TCGA Class1	2	0	0	0	0	0	0
	METABRIC Class 1	2	0	3	0	0	0	0
	METABRIC Class 2	8	0	0	0	0	1	0
Basal-like vs Her2	TCGA Class1	6	0	0	0	0	1	0
	TCGA Class3	4	0	0	0	0	1	0
	METABRIC Class 2	6	0	1	0	0	0	0

**Table 3:** Occurrence of the critical 3-node motifs enriched in Basal-specific networks. Motif type is described in the bracket.

	Basal-like vs Luminal A		Basal-like vs Luminal B			Basal-like vs Her2		
	TCGA class 1	METABRIC class 1	TCGA class1	METABRIC class 1	METABRIC class 2	TCGA class 1	TCGA class 3	METABRIC class 2
JUN-FOS-NR4A2	Present (linear, 021)	Present (linear, 021)	Present (linear, 021)	Absent	Present (linear, 021)	Present (linear, 021)	Absent	Present (linear, 021)
PML-STAT1-GCH1	Present (linear, 021)	Present (linear, 021)	Present (linear, 021)	Absent	Present (linear, 021)	Present (linear, 021)	Absent	Present (linear, 021)
STAT1-LCK-NR1H3	Present (linear, 021)	Present (complete, 030)	Absent	STAT1-NR1H3-LCK (linear, 021)	Present (complete, 030)	Absent	Present (complete, 030)	Present (linear, 021)

Both motifs JUN-FOS-NR4A2 and PML-STAT1-GCH1 form a 021 linear topology and were found in six out of eight networks. The motifs were absent from METABRIC class 1 from the Basal-like vs Luminal B comparison and from TCGA class 2 from the Basal-like vs Her2 comparison. Importantly, both classes were insubstantial *Basal dominant classes* from the corresponding comparisons.

Motif STAT1-LCK-NR1H3 forms a positive linear topology (type 021) and positive complete topology (type 030) in two and three Basal-specific networks, respectively. It must be noted that the 030 coherent (positive) complete motif represents a positive coordination between the three gene expressions, suggesting a tight positive transcriptional regulation. The same group of nodes in different arrangements are seen with STAT1, LCK and NR1H3 forming a STAT1-NR1H3-LCK 021 motif in the METABRIC class 1 from the Basal-like vs Luminal B comparison. The inconsistency of motif type observed with STAT1, LCK and NR1H3 could be caused by our choice of 1% threshold on the partial correlation networks. This could mean that an edge between STAT1, LCK and NR1H3 that fell out of this range could be excluded from the network, resulting in a change of the network motif topology (eg from type 030 to type 021). The 1% thresholding was found empirically, assigned after testing a series of threshold levels.

In summary, however, the correlations revealed here from our networks could be directly related to mRNA-mRNA interactions or could be an indication of protein-protein interactions (PPIs).

## Discussion

This work used a *Dirichlet* Process clustering to stratify breast cancer subtypes based on nuclear receptor expression from two independent breast cancer cohorts. The clustering uses a probabilistic approach to model the hierarchical organisation of the data according to its latent or natural structure [36], and therefore is expected to reveal a less biased stratification [35]. The *Basal dominant classes* identified in this work are distinct groups of patients that represent the Basal-like breast cancer subtype. To identify unique characteristics for Basal-like at the transcription system level, partial correlation networks were developed for each of the *Basal-dominant classes*. From the Basal-specific networks, hubs and the hub-associated local networks were identified. Three 3-node undirected network motifs were found to be consistent and enriched in the Basal-specific networks derived from the two datasets, representing critical network modules in Basal-like tumours. The consistency highlights the robustness of the observed patterns. While conventional drug target identification studies reveal individual candidates for targeting, this approach provides a system-level understanding of the hub NR genes [40] and their co-regulators. This partial correlation approach can help in identifying distinct and critical biological processes associated with the hub NRs and co-regulators implicated in Basal-like breast cancer development.

### Heterogeneity in Basal-like breast cancer

Based on the NR expression patterns, our clustering results are consistent with the PAM50 subtype classification [10]. In particular, the majority of Basal-like patients have distinct NR expression patterns compared to Luminal A and Luminal B patients. Surprisingly, in both the TCGA and METABRIC datasets used, a fraction of Basal-like patients (43/89 from TCGA, 46/199 from METABRIC) were shown to share more similarity with Her2 patients than to other Basal-like patients. This supports the well-recognised hypothesis that Basal-like subtype defined by the PAM50 sub-typing scheme is based on molecular heterogeneity [41–43]. While the PAM50 classification developed by Parker *et al.* has provided a foundation for breast cancer categorisation, most attention has been focussed on refining breast cancer subtypes [43–45]. For example, Sabatier *et al.* used a 368-gene expression signature to classify Basal-like breast cancer patients into two prognostic subgroups, with one group having better clinical outcomes than the other [46]. At the cellular level, Basal-like cell lines are classified into Basal-A and Basal-B, with Basal-A being more Luminal-like and Basal-B more Basal-like [44, 45]. Sotiriou *et al.* revealed a similar findings to this work using an unsupervised clustering from the statistical package BRB-ARRAYTOOLS software, observing the distinctive characteristics only between Basal-like and Luminal subtypes. Their clustering results revealed two natural Basal-like subgroups (Basal-1 and Basal-2), in which Basal-1 showed more similar gene expression signatures to Her2 compared to Basal-2 [47]. The Basal heterogeneity observed from Sotiriou *et al.* and in this work might reflect different cell origins, mutations or a combination of the two. Identifying factors that drive the Basal-like subtype diversity will be the next challenge. Importantly, while Sotiriou *et al.* revealed distinct Basal-like subgroups by analysing the whole genome pattern (7,650 probes), we obtained a similar stratification by using a much smaller and functionally focused subset of genes (NRs and co-regulators). Moreover, our approach using pairwise comparisons of Basal-like with other PAM50 breast cancer subtypes reveals similar or distinct characteristics within and between subtypes, providing insight into the different heterogeneous breast cancers.

Furthermore, it is important to recognise the presence of ambiguous cases (patients that fall into either an ambiguous class or group with other subtypes in our analyses), as they represent tumours with a greater heterogeneity and are often more difficult to treat [48]. A major challenge in understanding these ambiguous cases is that the sample size of these cases is often too small to establish sufficient detail. While the motifs identified in this work do not give indications of survival, or prediction measures for patients’ clinical outcome, a complementary analysis pursuing the second clustering strategy is discussed in S3.1 File in S3 Appendix. This second clustering approach uses the transform of the dataset to cluster NR gene expressions according to the patient profiles, resulting in NR clusters with distinct expression patterns. Clustering according to a non-parametric multinomial *Dirichlet* process stratification (using for example R/BHC) opens up novel avenues of transcriptome research offering opportunities in a number of fields.

### Critical network modules enriched in Basal-like NR expression correlation networks

In the eight correlation networks derived from the *Basal dominant classes*, FOS and STAT1 are the two most connected nodes. They are topologically central in the networks with maximal number of informative connections, representing important positions in the networks. It is evidenced that both FOS and STAT1 are significantly involved in various type of cancers and are well-known oncoregulators (FOS reviewed in [49], and STAT1 reviewed in [50]). To further evaluate these two hubs, we identified 3-node network motifs around FOS and STAT1. Three 3-node motifs (JUN-FOS-NR4A2, PML-STAT1-GCH1 and STAT1-LCK-NR1H3) were found to be enriched in Basal-specific networks across the two patient cohort databases, representing the core modules in the networks.

The network motif JUN-FOS-NR4A2 was found in most Basal-specific networks apart from the METABRIC class1 from Basal-like vs Luminal A comparison (Fig 2, **Right**) and Her2-like *Basal dominant class* from TCGA (Basal-like vs Her2 comparison, class3, Fig 4, **Left**). This is a linear motif with FOS in the middle. This suggests that FOS acts as a mediator and is critical for the JUN and NR4A2 association. Sotiriou *et al.* observed a similar breast cancer stratification to this work and reported that Basal-2 (the Basal-like Basal group) showed higher expression of FOS compared to Basal-1 (the Her2-like Basal) and other breast cancer subtypes [47]. FOS is a proto-oncogene and has been identified as a survival predictor [51] and a driver for breast cancer metastasis formation [52]. At the genetic level, gene FOS, JUN and NR4A2 are all classified as immediate early genes (IEGs) [53]. IEGs are activated rapidly in response to external stimuli, and are found to be over expressed in many cancers, including breast cancer [54]. IEGs over expression is thought to be caused by unchecked, the constitutively active MAPK signalling pathway in cancers [55]. At the protein level, gene FOS codes for functional protein c-FOS that dimerises with c-JUN proteins to form transcription factor complex AP-1 promoting breast cancer growth [56]. The dimerisation of JUN and FOS could explain the strong correlation between JUN and FOS revealed from the co-expression networks. Gene NR4A2 codes for NURR1 (nuclear receptor related 1 protein), a steroid thyroid hormone retinoid receptor. In breast cancer, it has been suggested that NR4A2 has a dichotomous role [57]. Although, currently no literature has provided a clear explanation for the FOS-NR4A2 correlation. Individually, FOS and NR4A2 have been identified as potential drug targets and biomarkers for breast cancer [57–59].

In this work, two network motifs were associated with STAT1, the other hub from the Basal-specific networks. Both STAT1 motifs are made up with genes that are involved in immune responses or in associated regulatory events. Specifically, in the PML-STAT1-GCH1 motif, all three genes have been shown to have an important role in the IFN*γ*-related defence response [60]. This result is in agreement with a recent study done by Thorsson *et al.* where they showed 60.5% Basal-like breast cancer displayed an IFN*γ* immune response signature, whereas only 22.8% of Luminal A, 46.6% of Luminal B, 49.3% of Her2 and 33.3% of normal-like patients displayed this signature [61]. IFN*γ* is an interferon produced by T helper cells (specifically, Th1 cells), cytotoxic T cells and macrophages in response to cytokine and antigen stimulation. Results from Thorsson *et al.* suggested that the tumour microenvironment of Basal-like breast cancer may be composed of a higher proportion of immune cells, cytokines and stroma than other breast cancer subtypes. This could explain the strong associations among the immune-related genes PML, STAT1 and GCH1 in Basal-like samples revealed in this study. Previous studies have also observed PML-STAT1 and STAT1-GCH1 associations, independently. For example, it has also been evidenced by Hsu *et al.* in preclinical models that PML can positively regulate STAT1/2 isgylation and transcriptional activity [62]. Moreover, previous studies have also demonstrated that the transcription of the GCH1 gene is mediated by the JAK2/STAT1 pathway [63] and positively regulated by STAT1 [64].

Expression of STAT1 has also been shown to correlate with LCK and NR1H3. While the STAT1-LCK and STAT1-NR1H3 correlations have not been reported by previous studies, it is possible that STAT1-LCK and STAT1-NR1H3 correlations represent immune-related cellular communications and cross-talks between signalling pathways. Specifically, mRNA expression of LCK codes for lymphocyte-specific protein tyrosine kinase which is a key kinase in T cells. Activated T cells produce interferons, including type I interferons (IFN-a, IFN-b). Type I interferons can bind to receptor proteins and activate JAK/STAT signalling pathway, regulating a series of downstream proteins, including STAT1 [65]. Moreover, while NH1H3 (LXR*α*) is not generally considered to be involved in immune responses or associated pathways, results from Pascual-García et al. have suggested that there is a cross-talk between IFN*γ*/STAT1 and LXRs (LXR*α* and LXR*β*) [66]. However, whether the STAT1-NR1H3 correlation revealed from this work represents a cross-talk between STAT1 and LXRs in Basal-like tumour needs further evaluation. Nevertheless, the two STAT1-associated motifs revealed from this study suggested that the tumour microenvironment of Basal-like breast cancer composed of immune cells and understanding the roles of immune system in Basal-like pathogenesis will be the next challenge.

As a concluding note, NR transcriptional networks reflect only a fraction of the system-wide interactome [26, 67]. This work not only revealed critical NR genes (hubs) implicated in the Basal-like subtype, but also examined the properties of these genes in the context of partial correlation networks. Most members of the three 3-node motifs identified from this work have been related to breast cancer oncogenesis, highlighting the relevance of these motifs in relation to Basal-like breast cancer. Compared to individual genes or biomarkers, hub-enriched biological modules as small network motifs could represent more robust prognostic signatures and provide a more meaningful context for further investigation, such as identifying pathways to target. The Basal-like NR 3-node motifs established here could be considered using enrichment analysis. This could further identify upstream regulators critical to these motifs [68]. We demonstrate enumeration of the nuclear receptor 3-node motifs of interest in Basal-like breast cancer and it is important to determine whether the same core modules can be recapitulated from other breast cancer patient cohort datasets. Finally, the utility of our method is not only restricted to Basal-like breast cancer but, of course, can be applied to other more complex cancers.

## Supporting information

S1 Appendix Definitions. This file contains detailed explanations of the specific terms used throughout this article.
(ZIP)

S2 Appendix NR-associated genes used in TCGA and METABRIC. 171 NR-associated genes were considered in the TCGA analyses. 169 NR-associated genes were considered in the METABRIC analyses.
(ZIP)

S3 Appendix Complete BHC results. This contains six sub-folders, including: **S3.1** explains the two-dimensional clustering analysis that can be performed using the patient-expression data. **S3.2** describes the process of consolidating clusters to classes. This was performed to reduce the number of clusters and to increase the sample size within a class. **S3.3** contains all the plots produced from this work, including three from TCGA and three from METABRIC. The blue solid lines in the dendrograms show preferred merges by BHC (clusters). Red dashed line show merges further up the cluster hierarchy. The numbers on the branches are the log odds for merging. **S3.4** contains the clustering results from the three TCGA analyses and an .pdf image illustrating a Venn diagram with the overlaps between the different TCGA *Basal dominant classes*. Each pairwise analysis is organised into its analysis folder, containing a *Data* folder (for the input files) and a *Working_directory* folder (for the output files). The *Data* folder contains a relevant median-centred patient expression data (.csv file). The *Working_directory* folder contains two text files (.txt) describing the members of patients or genes in the resulting clusters, two resulting plots (.pdf files) and R data (.RDa files) produced while running the code (provided in S3.6). Each analysis folder also contains a patients_hc.pdf file that illustrates the hierarchical structure for the patient clusters. This was generated separately for visualisation purposes using the plot() function and *hc_b.Rda* is in the *Working_directory* as the data. **S3.5** contains clustering results from the three METABRIC analyses. The folder organisations and files contained in this supplementary are equivalent to S3.4 above. **S3.6** contains the Clustering_code_using_BHC.R with written descriptions and remarks in code, provided for reproducible research.
(ZIP)

S4 Appendix Basal-specific networks derived from *Basal dominant classes* from TCGA and METABRIC. (PDF)

S5 Appendix Hub-associated local networks extracted from Basal-specific networks. This contains hub-associated local networks centred around FOS and STAT1 from the eight Basal-specific correlation networks. The local networks are defined by 3-node motifs, containing two levels of edges from the hubs. Hub genes are coloured in yellow and genes associated with hubs are coloured in blue. Positive edges (positive correlations) are in red and negative edges (negative correlations) are in black.
(PDF)

S6 Appendix Summary of node degree from Basal-specific networks. This summarises the number of degrees of each node across the eight Basal-specific networks.
(XLSX)

S7 Appendix Highlight of the three 3-node motifs embedded in Basal-specific networks. The 3-node network motifs that are enriched in Basal-like are highlighted in the Basal-specific networks. Hub genes are coloured in yellow, non-hub genes are coloured in blue. Correlation between nodes are shown in red lines. Numbers on the lines indicate the strength of the partial correlations.
(PDF)
