# Correction: Revealing nuclear receptor hub modules from Basal-like breast cancer expression networks

**DOI:** 10.1371/journal.pone.0314424

**Published:** 2024-11-19

**Authors:** Sharon Nienyun Hsu, Erika Wong En Hui, Mengzhen Liu, Di Wu, Thomas A. Hughes, James Smith

The ORCID iDs are missing for the second, third, fourth, fifth and sixth authors. Please see the authors’ respective ORCID ids here:

Author Erika Wong En Hui’s ORCID id is: 0000-0002-4283-3911

(https://orcid.org/0000-0002-4283-3911).

Author Mengzhen Liu’s ORCID id is: 0000-0003-2254-4204

(https://orcid.org/0000-0003-2254-4204).

Author Di Wu’s ORCID id is 0000-0001-8455-0349

(https://orcid.org/0000-0001-8455-0349).

Author Thomas A. Hughes’s ORCID id is 0000-0003-1169-3386

(https://orcid.org/0000-0003-1169-3386).

Author James Smith’s ORCID iD is 0000-0002-7465-6067

(https://orcid.org/0000-0002-7465-6067).
