# Supplementary material for: Revealing nuclear receptor hub modules from Basal-like breast cancer expression networks
Source: PLoS One. 2021 Jun 23;16(6):e0252901. doi: 10.1371/journal.pone.0252901 (PMC8221501; doi:10.1371/journal.pone.0252901)
Supplement: S1 Appendix — This file contains detailed explanations of the specific terms used throughout this article. (ZIP) [file pone.0252901.s001.zip › S_1_Appendix - Definitions/S_1_definitions_Table.pdf]

SI 1 Definitions

**Abbreviations/Terminology**	**Description**
BC	breast cancer
TNBC	triple negative breast cancer
ER	estrogen receptor
PGR	progesterone receptor
Her2	human epidermal growth factor receptor 2
GCN	gene correlation network
Cluster	Clusters are the initial grouppings revealed by BHC.
Class	Classes are consolidated from clusters based on separation further up the cluster hierarchy (shown in the result dendrogram). Although it is a simplification of the clusters, classes still represent distinct groups.
Subtype dominant class	A patient class that is dominated by a certain breast cancer subtype.
Ambiguous class	A patient class that is not dominated by any breast cancer subtype and contains similar amount of patients from both subtypes.
Basal dominant class	A patient class that is dominated by Basal subtype. *Basal dominant classes* are the focus of this study and are further examined. In this study, we have identified eight *Basal dominant classes* from three comparisons and two patient cohorts.
NR-associated genes	Hundred and seventy eight NR-associated genes were considered in this study, including 46 nuclear receptors (NRs) and 132 NR co-regulators. The 132 NR co-regulators are the mRNAs that code for proteins that an association with any of the 46 NRs. The NR co-regulators are described in the STRING database. Seven NR co-regulators were not available in TCGA, therefore only 171 NR-associated genes (46 NRs + 125 co-regulators) were considered in the TCGA analyses. Nine NR co-regulators were not available in METABRIC, therefor only 169 NR-associated genes (46 NRs + 123 co-regulators) were considered in the METABRIC analyses.
Basal-specific network	Basal-specific networks are the undirected partial correlation networks developed for *Basal dominant classes*. Eight Basal-specific networks were developed and explored in this study. In Basal-specific networks, all nodes are labelled with an NR identity and the relationship between the nodes can be positive (red) or negative (black).

**Abbreviations/Terminology**	**Description**
Hubs	In this study, the hubs are the top three most connected nodes in the eight Basal-specific networks. As a proof of concept, only three nodes with the maximal connections were considered, more effort is require to explore other nodes with high connectivity.
Edges	In correlation networks, edges are the lines between the nodes, representing a correlation between two variables. Correlations can be positive or negative and are shown in red and black in the networks, respectively.
3-node motifs	Three-node motifs are the smallest functional unit in a network graph. They are defined by three nodes (eg A, B, C) and three relationships (A->B, B->C and C->A). There are 27 possible 3-node motifs configurations from an undirected correlation networks.
Hub-associated local network	Hub-associated local networks are extracted Basal-specific networks. They are defined by 3-node motifs, including two levels of edges from the hubs.
NPU motif classification	The NPU motifs classification is adopted from the concept of triad census proposed by J. Davis and S. Leinhardt. For each 3-node motif, we counted the number of negative, positive and un-associated edges and assigned each motif with a three digital code. The first number indicates the number of negative edges, second number is the number of positive edges and the last number is the number if un-associated edges (no edges). All 27 possible 3-node configurations were classified into 10 groups. Each group can have more than one non-redundant configurations, if define each node a clockwise labelling, eg A, B, C (Fig6).
Linear topology	Linear topology is any three nodes with two edges, edges can be positive or negative. If all three nodes are given a clockwise labelling (eg A, B, C), there are 12 possible non-redundant configurations from undirected correlation networks. (see Fig7)
Complete topology	Complete topology is any three nodes with three edges, edges can be positive or negative. If all three nodes are given a clockwise labelling (eg A, B, C), there are 8 possible non-redundant configurations from undirected correlation networks. Four of them are coherent and the other four represent incoherent behaviour. (see Fig8)
