# Supplementary material for: Revealing nuclear receptor hub modules from Basal-like breast cancer expression networks
Source: PLoS One. 2021 Jun 23;16(6):e0252901. doi: 10.1371/journal.pone.0252901 (PMC8221501; doi:10.1371/journal.pone.0252901)
Supplement: S2 Appendix — 171 NR-associated genes were considered in the TCGA analyses. 169 NR-associated genes were considered in the METABRIC analyses. (ZIP) [file pone.0252901.s002.zip › S_2_Appendix - NR-associated genes used in TCGA and METABRIC/S_2_NR-associated genes used in TCGA and METABRIC.pdf]

SI 2. This supplementary table summarises the gene expression avaliable in TCGA and METABRIC dataset.
TCGA analysis considered 171 genes and is missing: AGO2, KAT2B, NR2C2AP, SGK1, SKP1, TAB2, VANGL2.
METABRIC analysis considered 169 genes and is missing: ADIPOQ, CYP1A2, CYP2B6, AGO2, HNF4G, INS. NANOG, SCNN1A, TCF3.

	TCGA	METABRIC
ABCA1	✓	✓
ABCB1	✓	✓
ABCB11	✓	✓
ABCG8	✓	✓
ADIPOQ	✓	x
AGO2	x	x
AKT1	✓	✓
APOA1	✓	✓
APOB	✓	✓
AR	✓	✓
ARNTL	✓	✓
BCL11A	✓	✓
BCL2	✓	✓
BCL6	✓	✓
BRCA1	✓	✓
CCND1	✓	✓
CDK2	✓	✓
CLOCK	✓	✓
COPS2	✓	✓
CREB1	✓	✓
CREBBP	✓	✓
CTNNB1	✓	✓
CYP19A1	✓	✓
CYP1A2	✓	x
CYP2B6	✓	x
CYP2E1	✓	✓
CYP3A4	✓	✓
CYP7A1	✓	✓
DR1	✓	✓
EP300	✓	✓
ERBB2	✓	✓
ESR1	✓	✓
ESR2	✓	✓
ESRRA	✓	✓
ESRRB	✓	✓
ESRRG	✓	✓
EWSR1	✓	✓
FABP1	✓	✓
FABP6	✓	✓
FKBP1A	✓	✓
FKBP1B	✓	✓
FKBP4	✓	✓
FOS	✓	✓
FOXL2	✓	✓
FOXO1	✓	✓
FOXP3	✓	✓
GCH1	✓	✓
GPS2	✓	✓
HDAC1	✓	✓
HDAC3	✓	✓
HIVEP1	✓	✓
HNF1A	✓	✓
HNF4A	✓	✓
HNF4G	✓	x
HSP90AA1	✓	✓
IKBKB	✓	✓
INS	✓	x
JAZF1	✓	✓
JUN	✓	✓
KAT2B	x	✓

KLF4	✓	✓
KLK3	✓	✓
LCK	✓	✓
LEP	✓	✓
LMX1A	✓	✓
LMX1B	✓	✓
LPL	✓	✓
MAPK1	✓	✓
MAPK8	✓	✓
MED1	✓	✓
MED24	✓	✓
MEF2D	✓	✓
NANOG	✓	x
NCOA1	✓	✓
NCOA2	✓	✓
NCOA3	✓	✓
NCOA6	✓	✓
NCOR1	✓	✓
NCOR2	✓	✓
NFKB1	✓	✓
NISCH	✓	✓
NOS3	✓	✓
NPAS2	✓	✓
NR0B1	✓	✓
NR0B2	✓	✓
NR1D1	✓	✓
NR1D2	✓	✓
NR1H2	✓	✓
NR1H3	✓	✓
NR1H4	✓	✓
NR1I2	✓	✓
NR1I3	✓	✓
NR2C1	✓	✓
NR2C2	✓	✓
NR2C2AP	x	✓
NR2E1	✓	✓
NR2E3	✓	✓
NR2F1	✓	✓
NR2F2	✓	✓
NR2F6	✓	✓
NR3C1	✓	✓
NR3C2	✓	✓
NR4A1	✓	✓
NR4A2	✓	✓
NR4A3	✓	✓
NR5A1	✓	✓
NR5A2	✓	✓
NRBP1	✓	✓
NRF1	✓	✓
NRIP1	✓	✓
NRP2	✓	✓
PAX6	✓	✓
PCK2	✓	✓
PGR	✓	✓
PITX3	✓	✓
PML	✓	✓
PNRC2	✓	✓
POMC	✓	✓
POU5F1	✓	✓
PPARA	✓	✓
PPARD	✓	✓
PPARG	✓	✓
PPARGC1A	✓	✓
PPARGC1B	✓	✓
PSMC1	✓	✓

PSMD2	√	√
RARA	√	√
RARB	√	√
RARG	√	√
RARS	√	√
RASD1	√	√
RELA	√	√
RERE	√	√
RGL2	√	√
RNF14	√	√
RORA	√	√
RORB	√	√
RORC	√	√
RPS27A	√	√
RXRA	√	√
RXRB	√	√
RXRG	√	√
SCNN1A	√	x
SGK1	x	√
SIRT1	√	√
SIX3	√	√
SKP1	x	√
SLCO1B1	√	√
SMAD3	√	√
SMAD4	√	√
SMARCA2	√	√
SMARCA4	√	√
SMARCD3	√	√
SOX2	√	√
SOX9	√	√
SP1	√	√
SRC	√	√
STAR	√	√
STAT1	√	√
STX17	√	√
SUMO1	√	√
TAB2	x	√
TAF15	√	√
TCF12	√	√
TCF3	√	x
TFG	√	√
THRA	√	√
THRB	√	√
THRSP	√	√
TMPRSS2	√	√
TRERF1	√	√
TRIM24	√	√
UBC	√	√
UBE2I	√	√
USF2	√	√
VANGL2	x	√
VDR	√	√
WT1	√	√
