# Supplementary material for: Revealing nuclear receptor hub modules from Basal-like breast cancer expression networks
Source: PLoS One. 2021 Jun 23;16(6):e0252901. doi: 10.1371/journal.pone.0252901 (PMC8221501; doi:10.1371/journal.pone.0252901)

Basal vs Luminal A from TCGA

classes	1	2	3	4	5
Basal	89	3	1	5	0
Luminal A	1	15	104	24	87

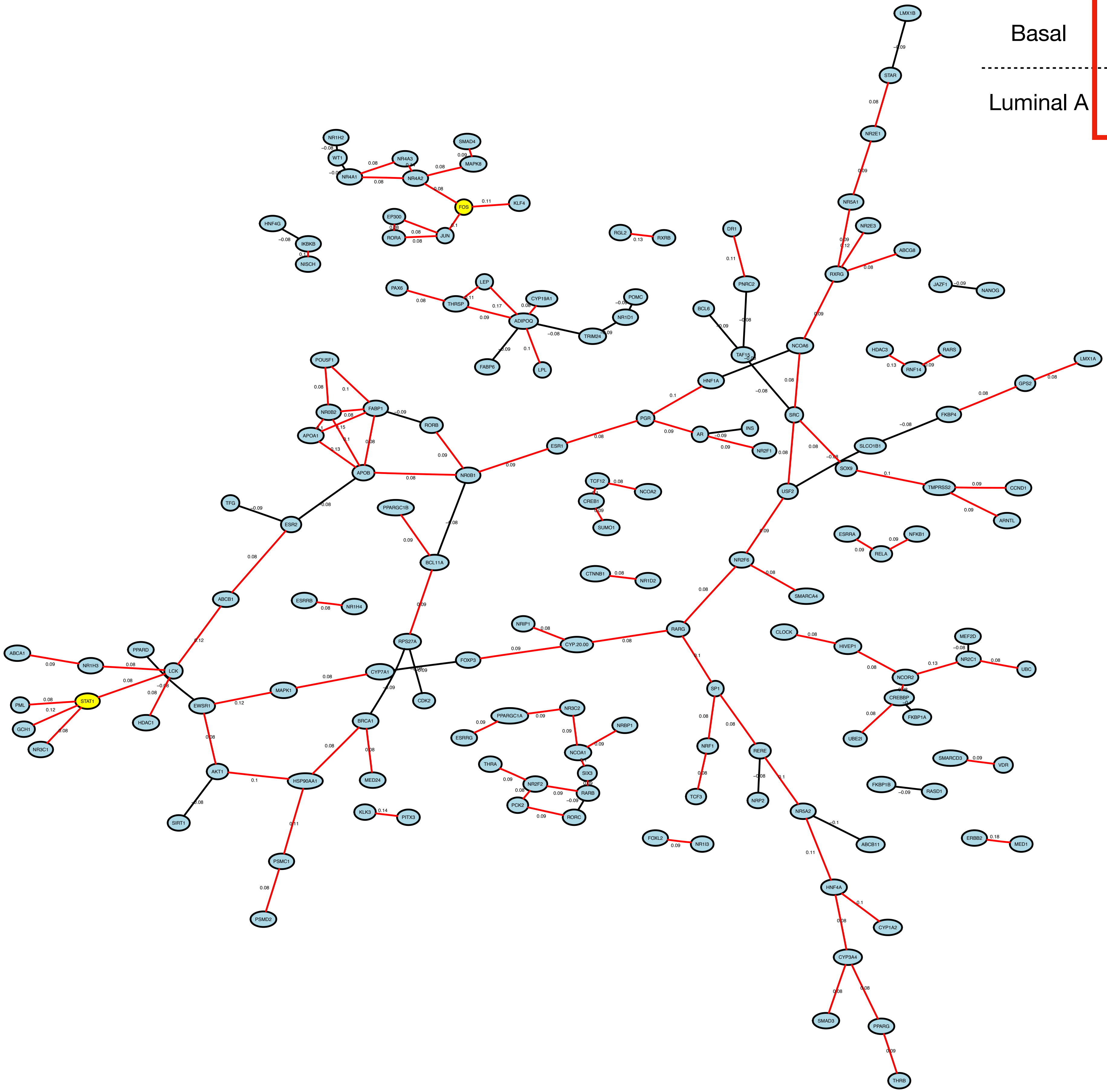

Basal vs Luminal A from METABRIC

classes	1	2	3	4	5	6
Basal	139	23	0	1	11	25
Luminal A	0	48	106	206	247	72

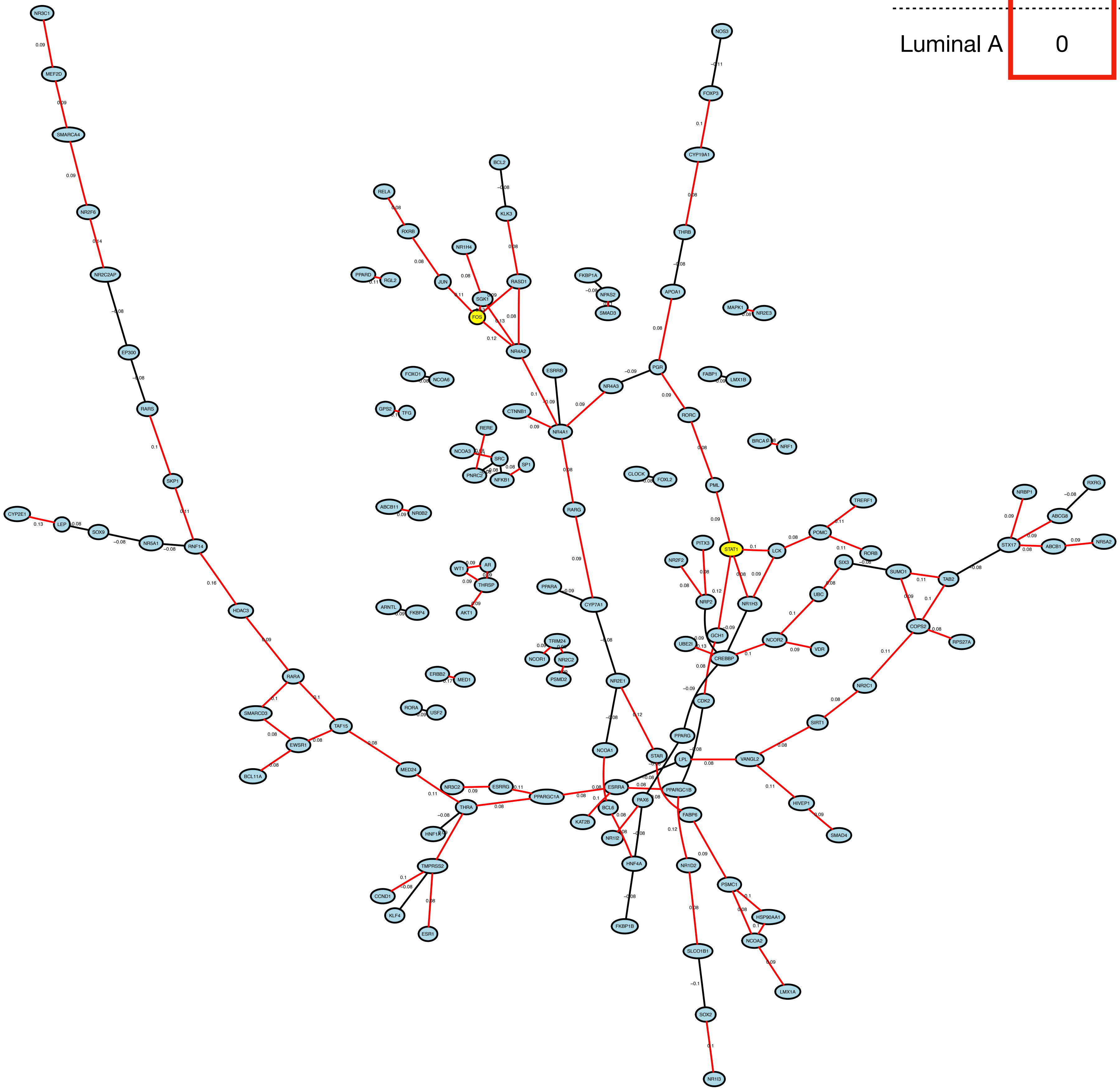

Basal vs Luminal B from TCGA

classes	1	2	3
Basal	80	17	1
Luminal B	5	32	90

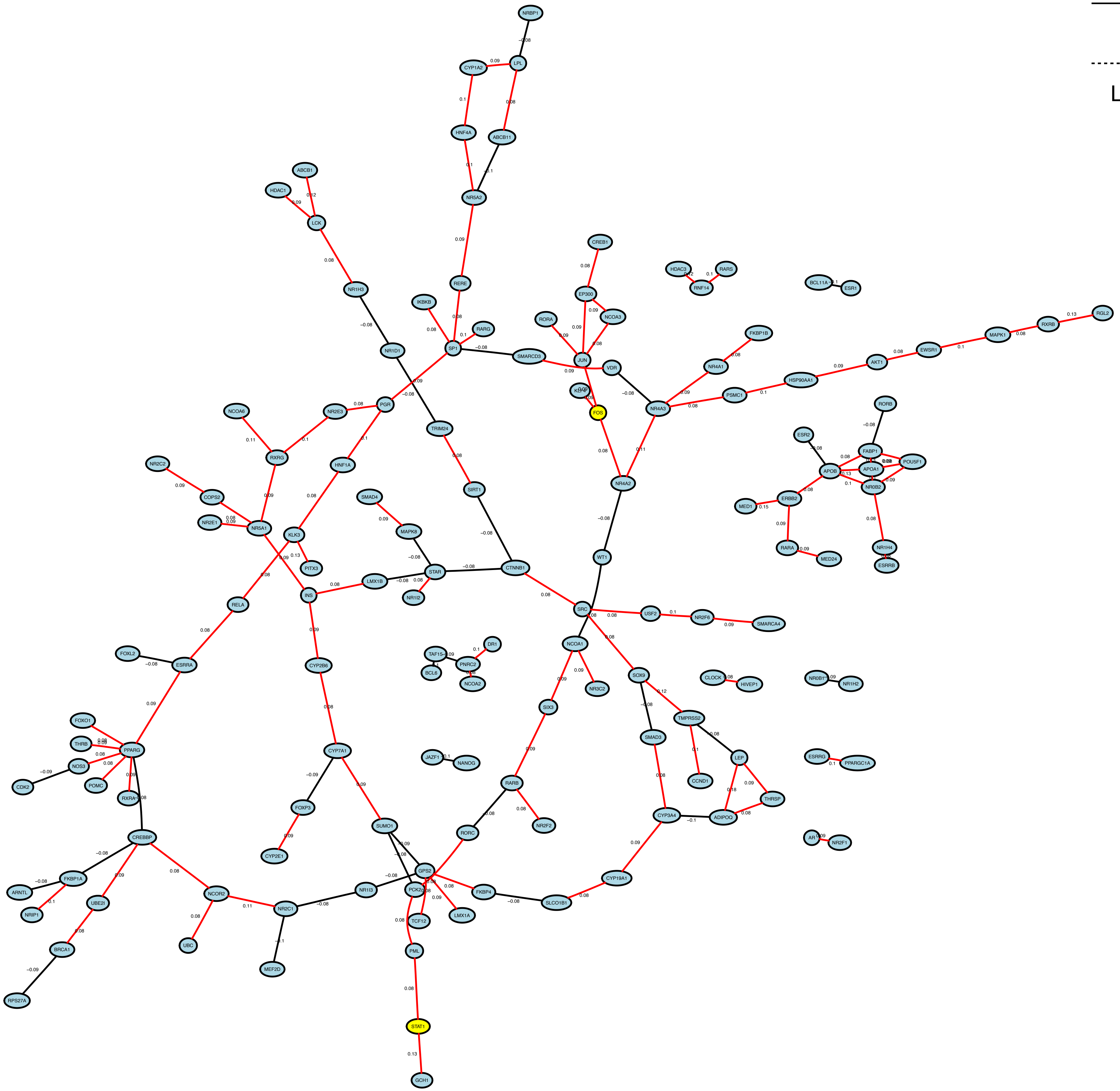

Basal vs Luminal B from METABRIC

classes	1	2	3	4	5	6	7
Basal	50	111	0	1	2	7	28
Luminal B	13	3	116	153	26	120	30

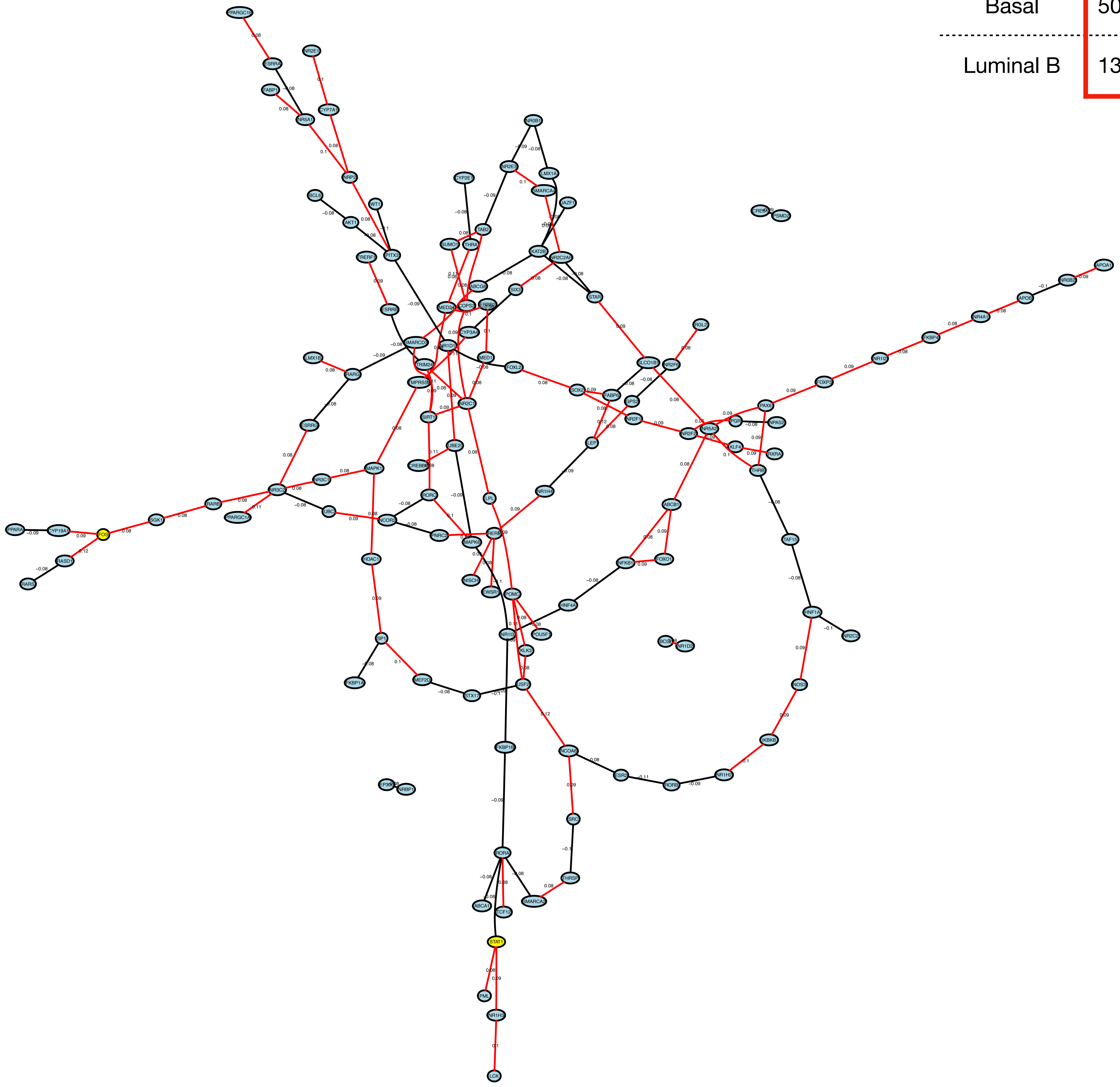

Basal vs Luminal B from METABRIC

classes	1	2	3	4	5	6	7
Basal	50	111	0	1	2	7	28
Luminal B	13	3	116	153	26	120	30

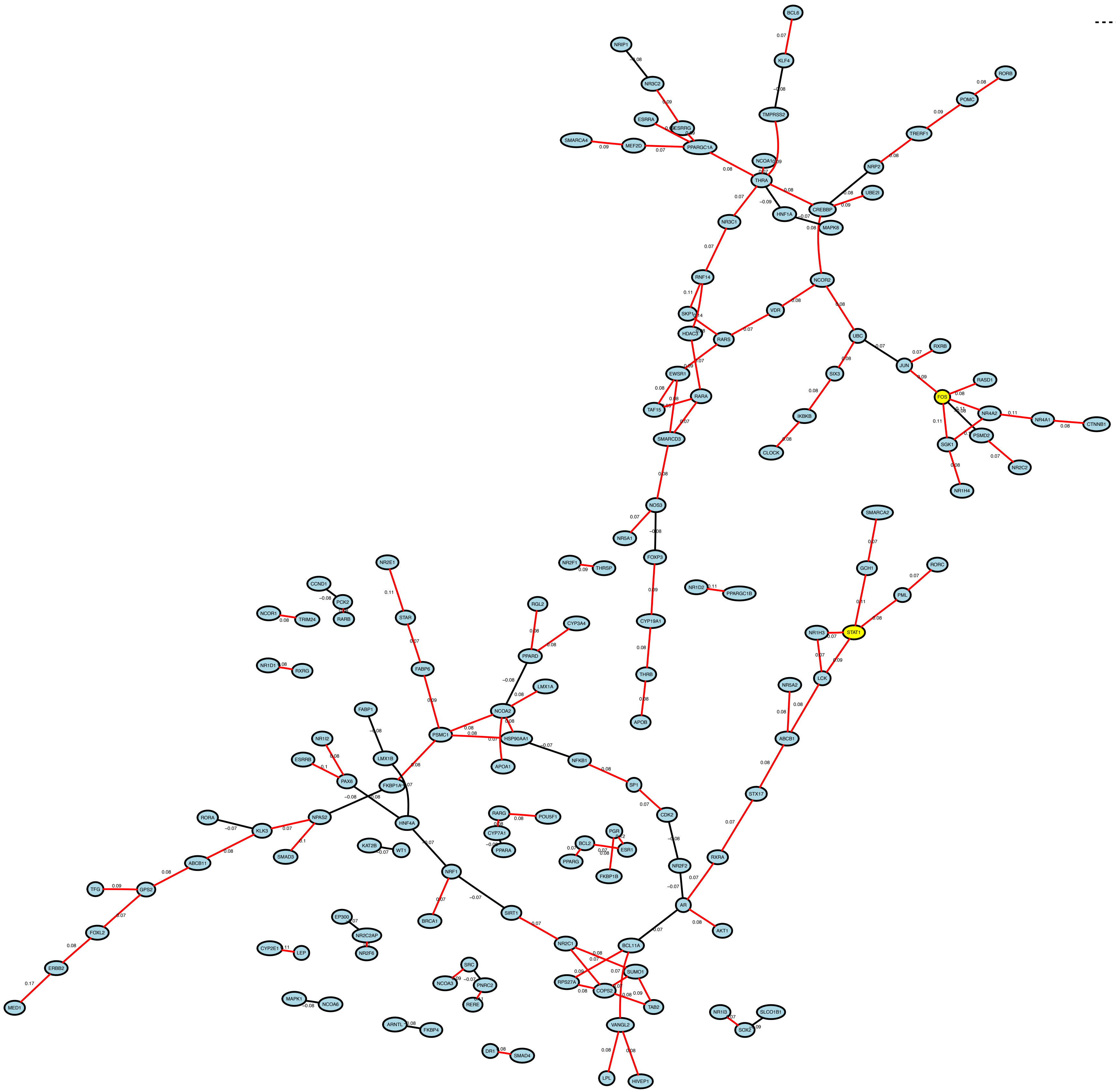

Basal vs Her2 from TCGA

classes	1	2	3	4
Basal	45	1	43	9
Her2	7	40	4	7

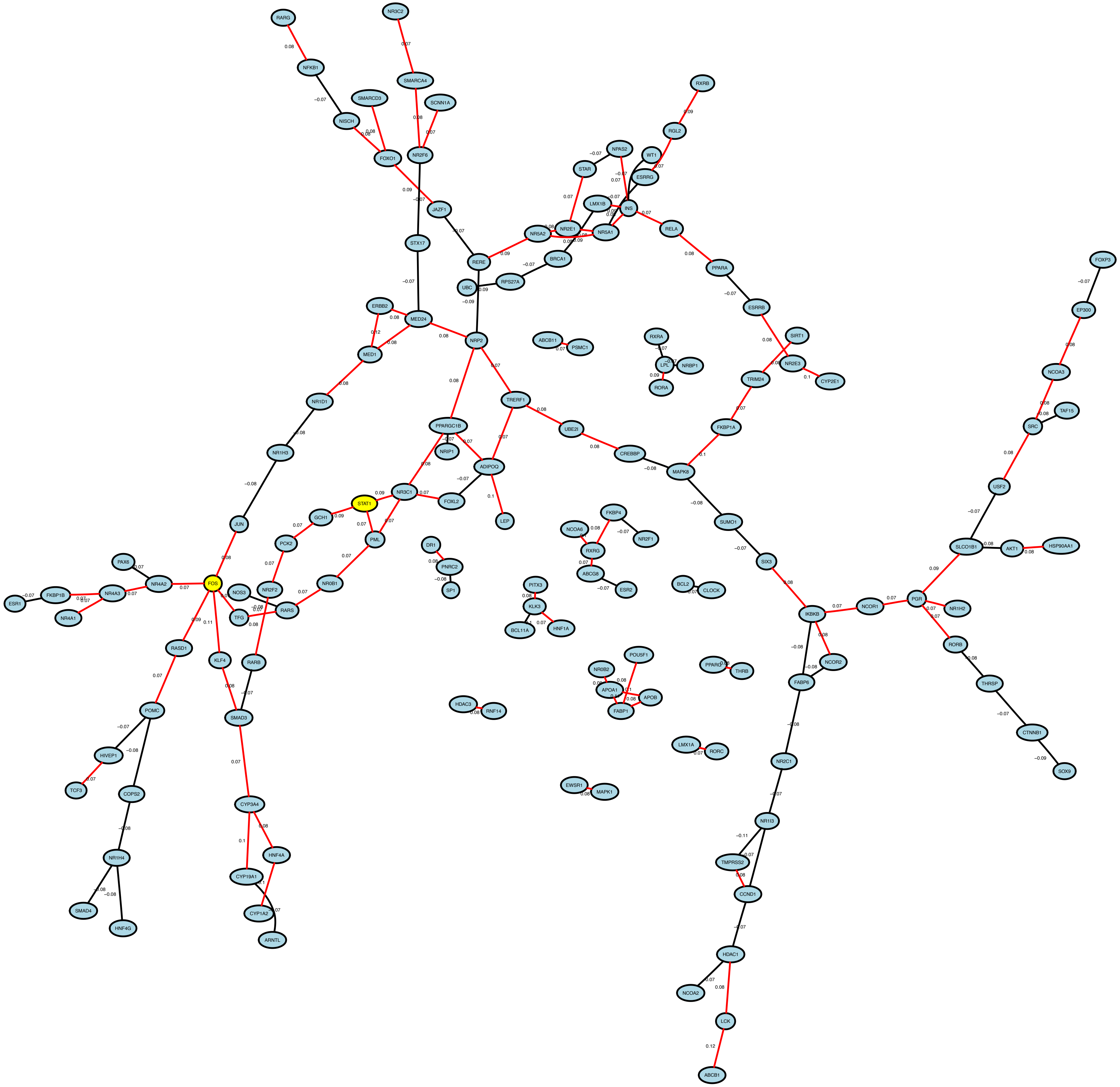

Basal vs Her2 from TCGA

classes	1	2	3	4
Basal	45	1	43	9
Her2	7	40	4	7

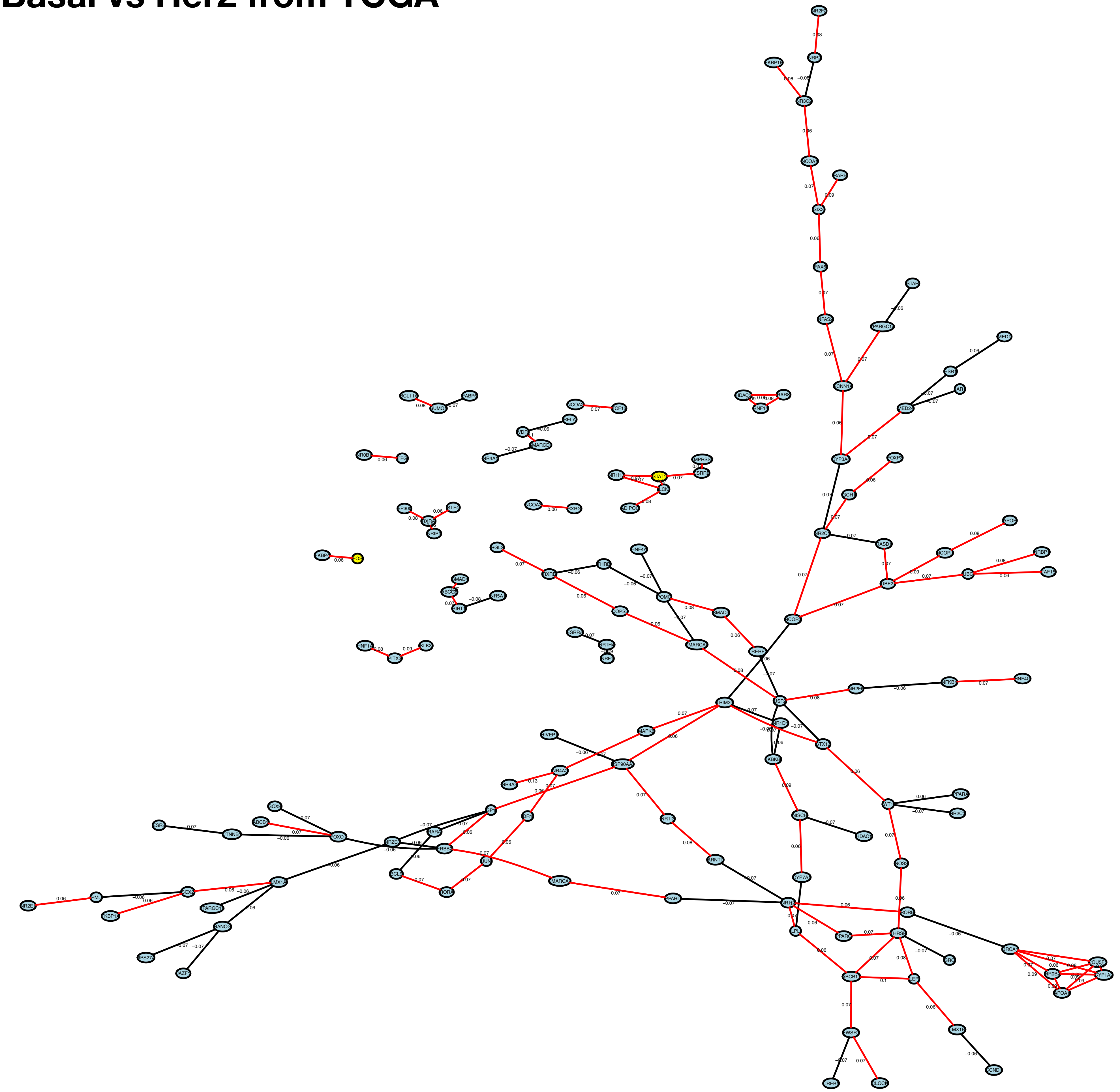

Basal vs Her2 from METABRIC

classes	1	2	3	4	5	6	7
Basal	11	115	46	3	4	7	13
Her2	32	1	130	15	17	9	16

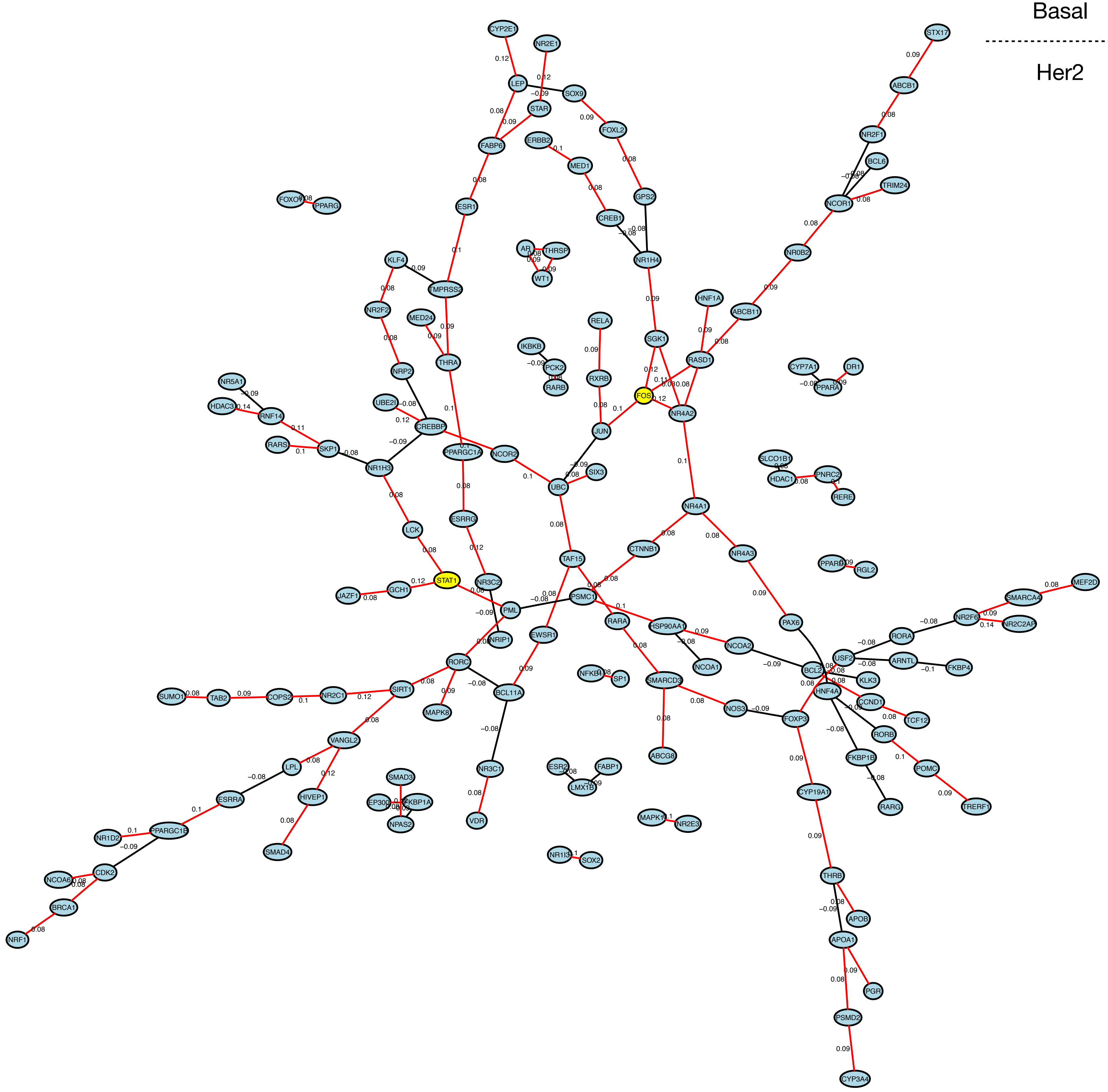

Supplement: S4 Appendix — (PDF) [file pone.0252901.s004.pdf]
