# Supplementary material for: Revealing nuclear receptor hub modules from Basal-like breast cancer expression networks
Source: PLoS One. 2021 Jun 23;16(6):e0252901. doi: 10.1371/journal.pone.0252901 (PMC8221501; doi:10.1371/journal.pone.0252901)
Supplement: S5 Appendix — This contains hub-associated local networks centred around FOS and STAT1 from the eight Basal-specific correlation networks. The local networks are defined by 3-node motifs, containing two levels of edges from the hubs. Hub genes are coloured in yellow and genes associated with hubs are coloured in blue. Positive edges (positive correlations) are in red and negative edges (negative correlations) are in black. (PDF) [file pone.0252901.s005.pdf]

SI 5. The hub-associated local networks centred around FOS and STAT1 from the eight Basal-specific correlation networks. The local networks are defined by 3-node motifs, containing two levels of edges from the hubs. Hub genes are coloured in yellow and genes associated with hubs are coloured in blue. Positive edges (correlation) are in red and negative edges are in black.

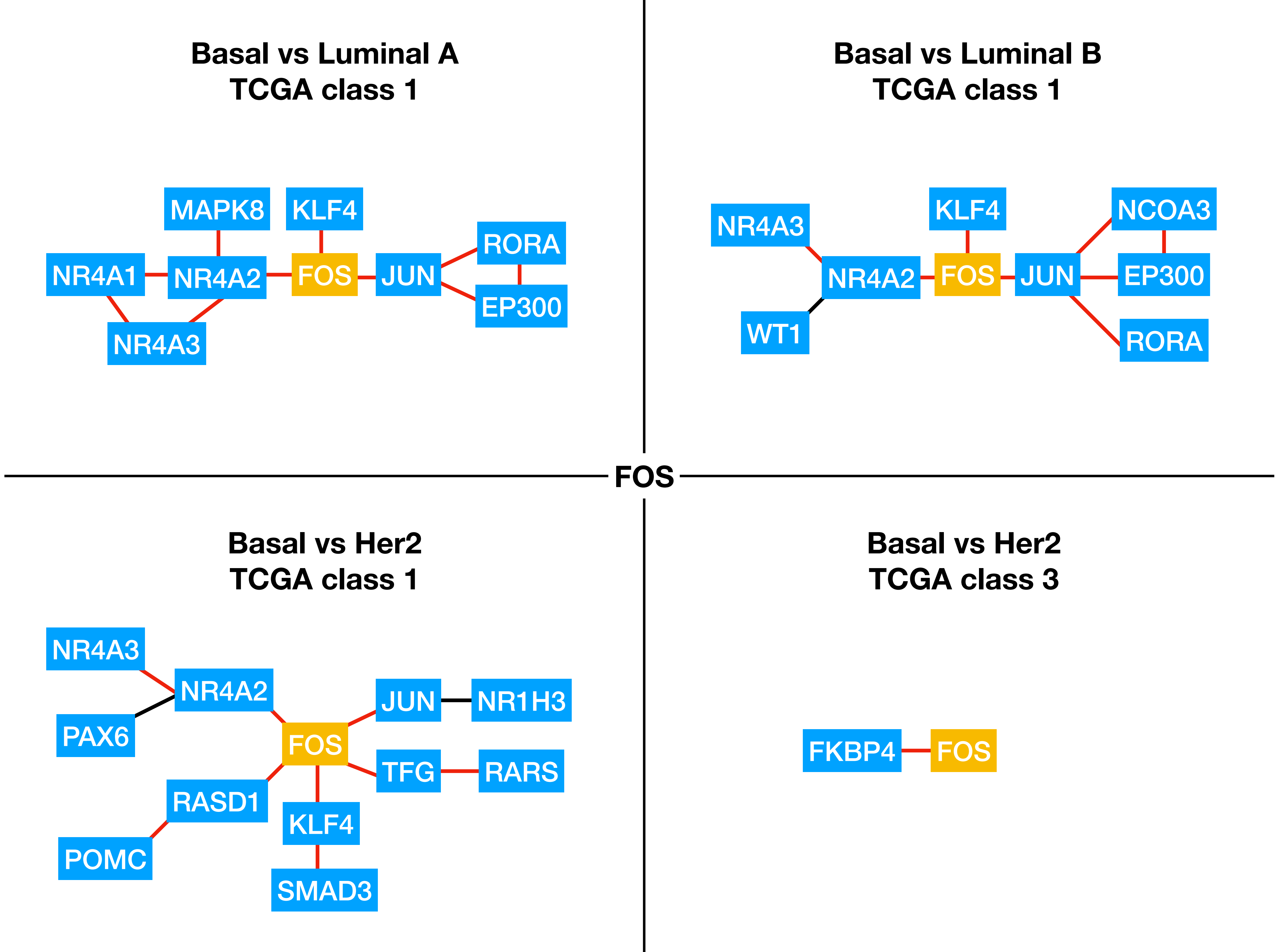
Basal vs Luminal A
TCGA class 1
MAPK8
KLF4
RORA
NR4A1
NR4A2
FOS
JUN
EP300
NR4A3
Basal vs Luminal B
TCGA class 1
NR4A3
KLF4
NCOA3
NR4A2
FOS
JUN
EP300
WT1
RORA
FOS
Basal vs Her2
TCGA class 1
NR4A3
NR4A2
PAX6
JUN
NR1H3
FOS
TFG
RARS
RASD1
KLF4
POMC
SMAD3
Basal vs Her2
TCGA class 3
FKBP4
FOS

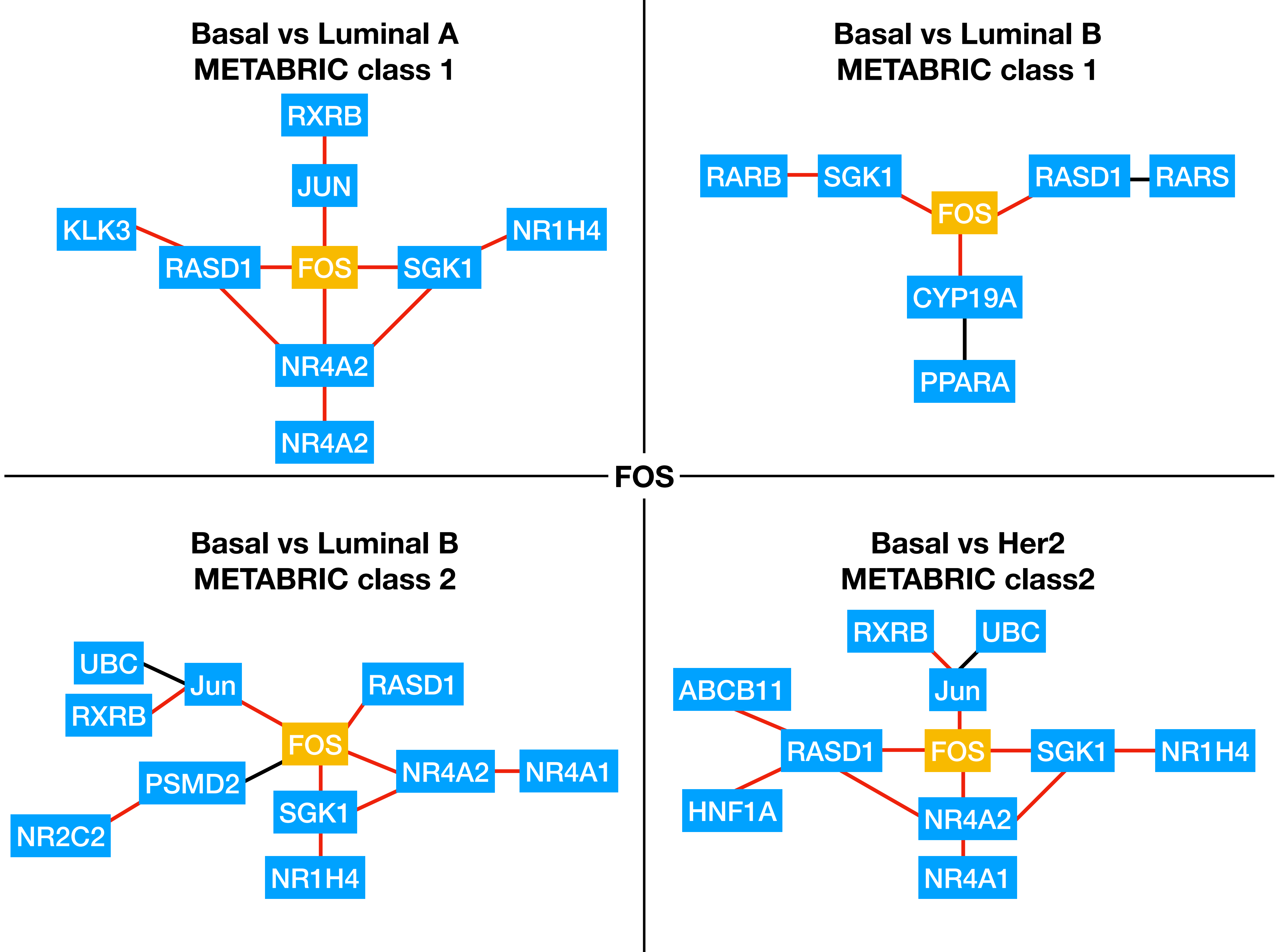
Basal vs Luminal A
METABRIC class 1
RXRB
JUN
KLK3
NR1H4
RASD1
FOS
SGK1
NR4A2
NR4A2
Basal vs Luminal B
METABRIC class 1
RARB
SGK1
RASD1
RARS
FOS
CYP19A
PPARA
FOS
Basal vs Luminal B
METABRIC class 2
UBC
Jun
RASD1
RXRB
FOS
PSMD2
NR4A2
NR4A1
SGK1
NR2C2
NR1H4
Basal vs Her2
METABRIC class2
RXRB
UBC
ABCB11
Jun
RASD1
FOS
SGK1
NR1H4
HNF1A
NR4A2
NR4A1

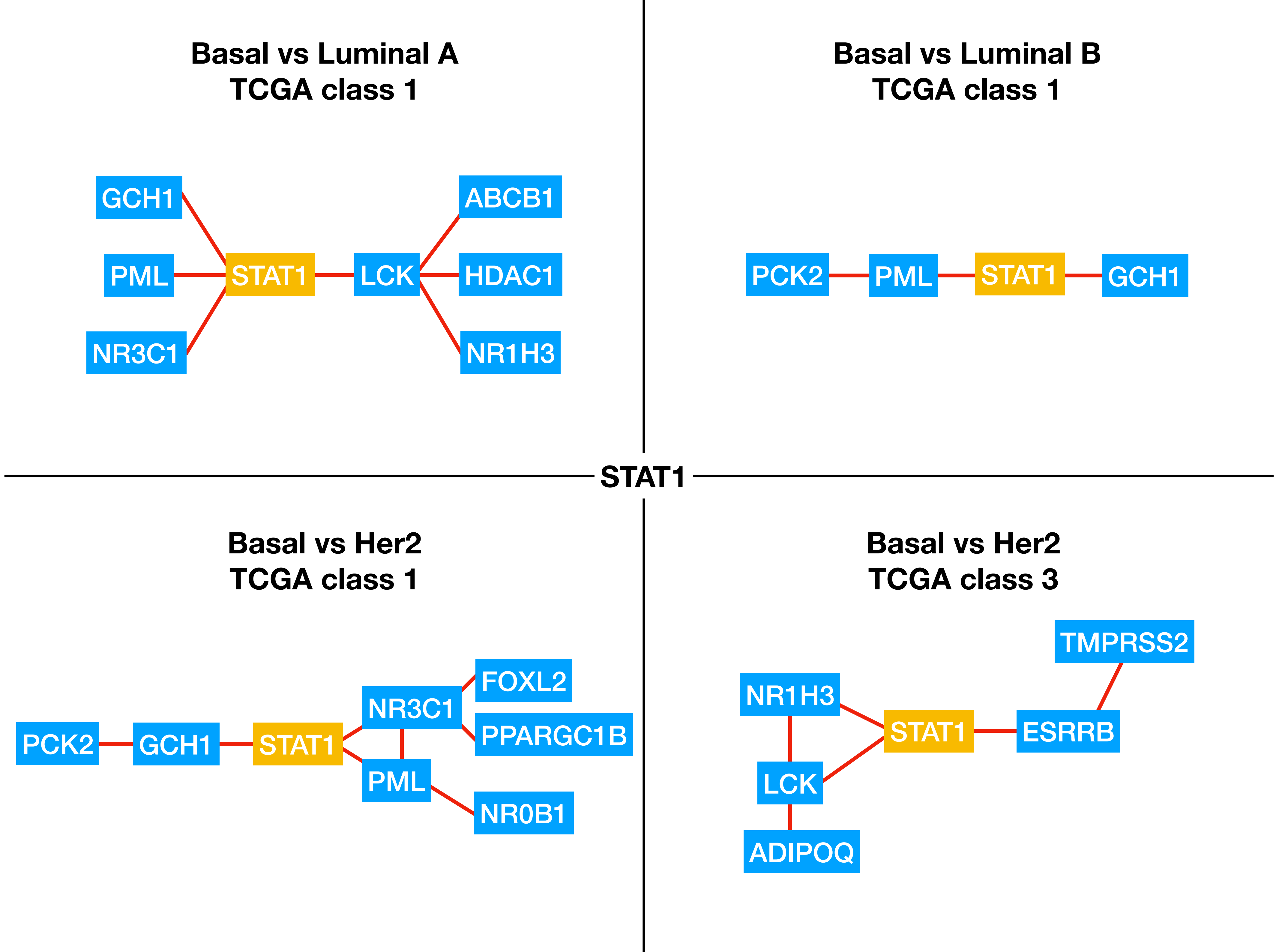

Basal vs Luminal A
TCGA class 1
GCH1
PML
NR3C1
STAT1
LCK
ABCB1
HDAC1
NR1H3
Basal vs Luminal B
TCGA class 1
PCK2
PML
STAT1
GCH1
STAT1
Basal vs Her2
TCGA class 1
PCK2
GCH1
STAT1
NR3C1
PML
FOXL2
PPARGC1B
NR0B1
Basal vs Her2
TCGA class 3
TMPRSS2
NR1H3
STAT1
ESRRB
LCK
ADIPOQ

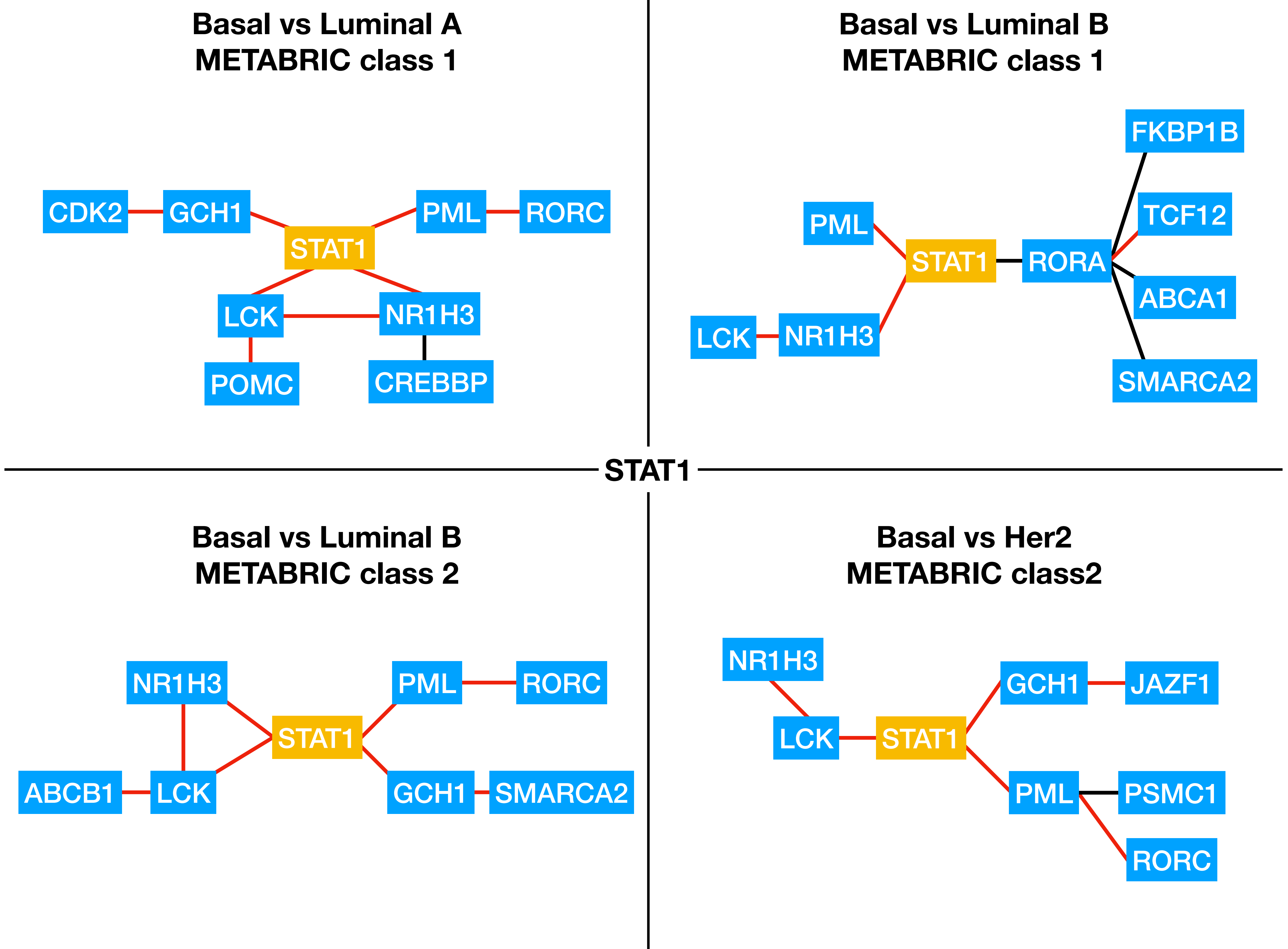
Basal vs Luminal A
METABRIC class 1
CDK2
GCH1
STAT1
PML
RORC
LCK
NR1H3
POMC
CREBBP
Basal vs Luminal B
METABRIC class 1
FKBP1B
TCF12
PML
STAT1
RORA
ABCA1
LCK
NR1H3
SMARCA2
STAT1
Basal vs Luminal B
METABRIC class 2
NR1H3
PML
RORC
STAT1
ABCB1
LCK
GCH1
SMARCA2
Basal vs Her2
METABRIC class2
NR1H3
GCH1
JAZF1
LCK
STAT1
PML
PSMC1
RORC
