# Supplementary material for: Revealing nuclear receptor hub modules from Basal-like breast cancer expression networks
Source: PLoS One. 2021 Jun 23;16(6):e0252901. doi: 10.1371/journal.pone.0252901 (PMC8221501; doi:10.1371/journal.pone.0252901)
Supplement: S7 Appendix — The 3-node network motifs that are enriched in Basal-like are highlighted in the Basal-specific networks. Hub genes are coloured in yellow, non-hub genes are coloured in blue. Correlation between nodes are shown in red lines. Numbers on the lines indicate the strength of the partial correlations. (PDF) [file pone.0252901.s007.pdf]

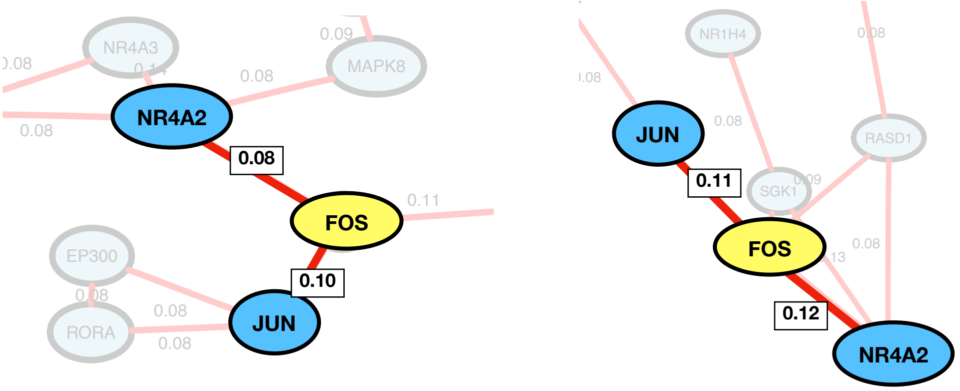

TCGA class1 METABRIC class1

Basal vs Luminal A

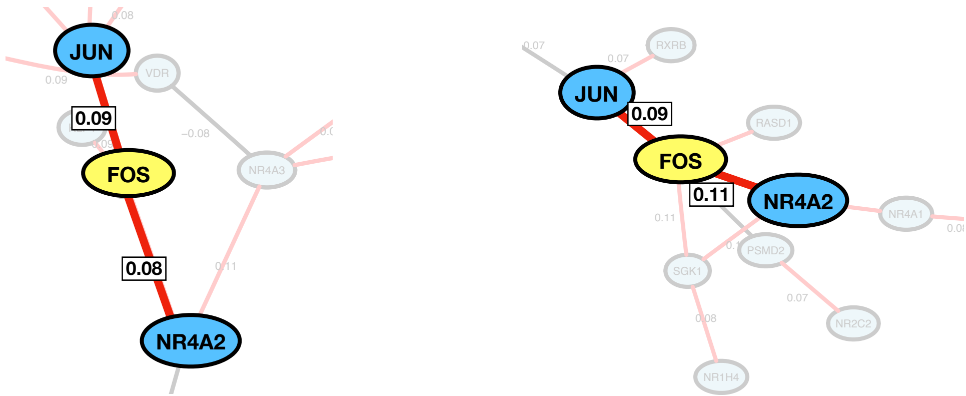

TCGA class 1 METABRIC class 2

Basal vs Luminal B

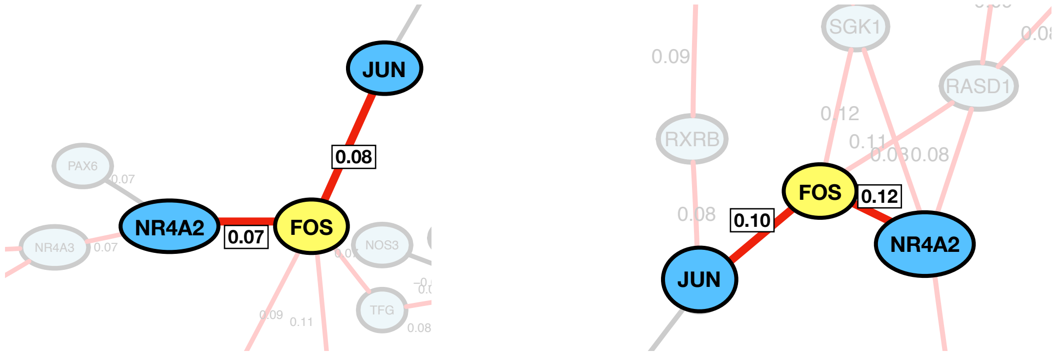

TCGA class 1 METABRIC class 2

Basal vs Her2

Motif JUN-FOS-NR4A2

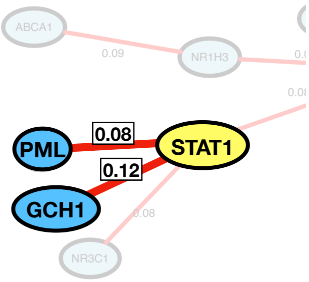

TCGA class1

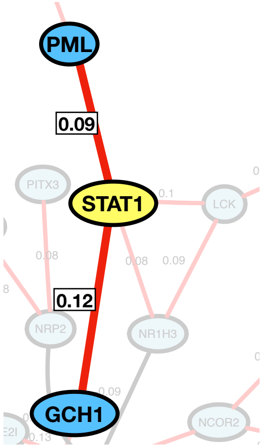

METABRIC class1

Basal vs Luminal A

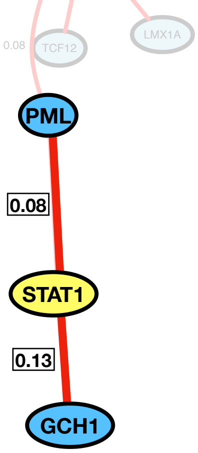

TCGA class 1

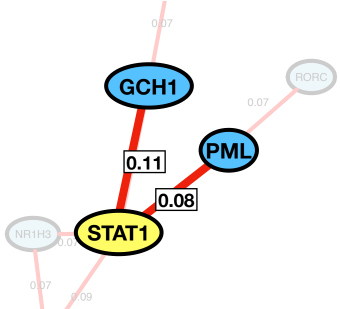

METABRIC class 2

Basal vs Luminal B

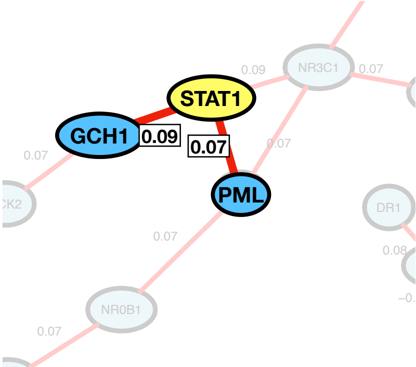

TCGA class 1

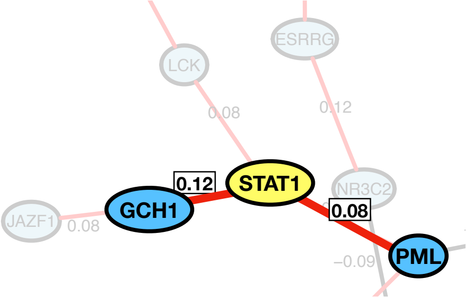

METABRIC class 2

Basal vs Her2

Motif PML-STAT1-GCH1

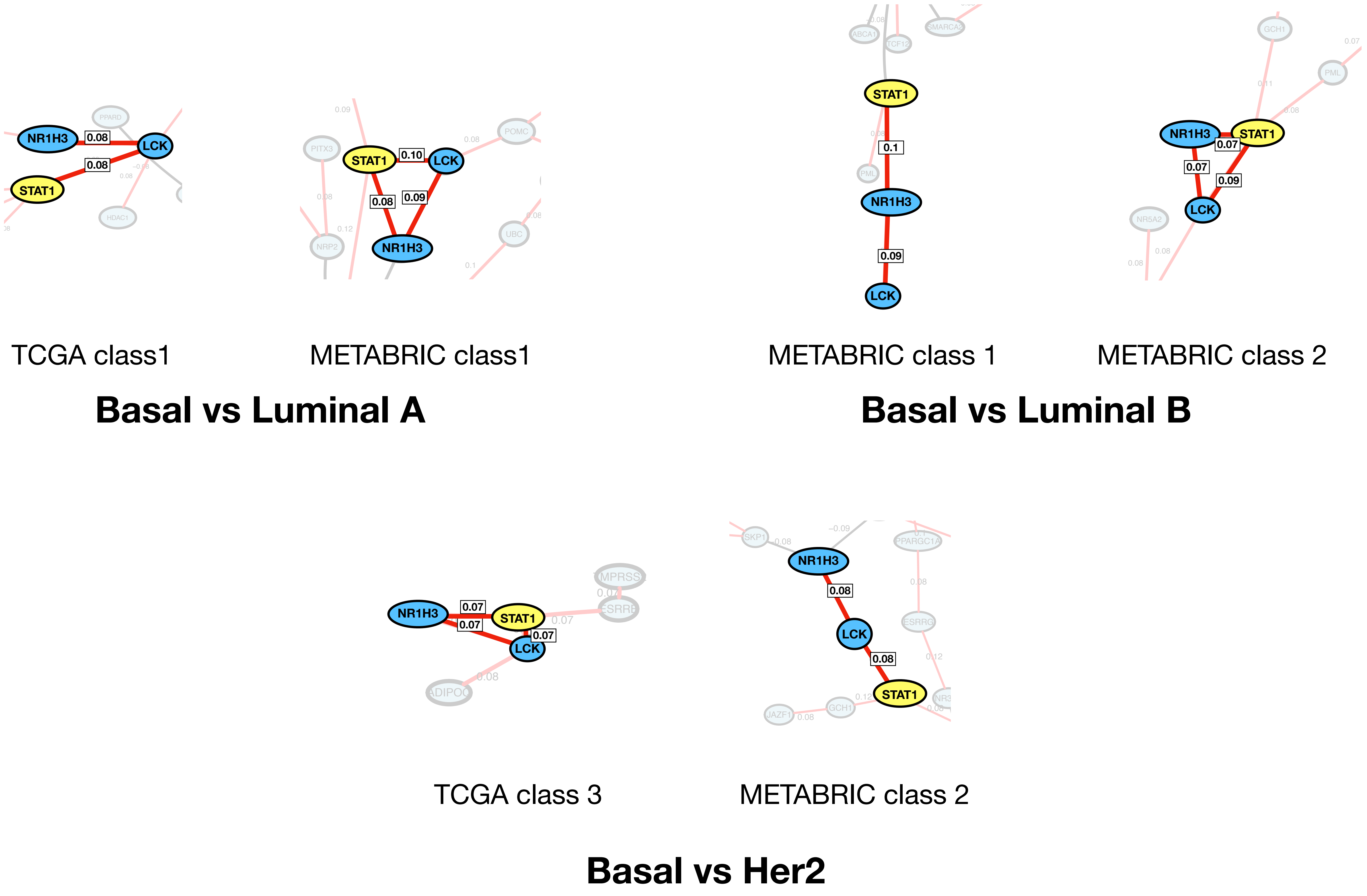

Motif STAT1-LCK-NR1H3
