# Supplementary material for: Revealing nuclear receptor hub modules from Basal-like breast cancer expression networks
Source: PLoS One. 2021 Jun 23;16(6):e0252901. doi: 10.1371/journal.pone.0252901 (PMC8221501; doi:10.1371/journal.pone.0252901)
Supplement: S3 Appendix — This contains six sub-folders, including: S3.1 explains the two-dimensional clustering analysis that can be performed using the patient-expression data. S3.2 describes the process of consolidating clusters to classes. This was performed to reduce the number of clusters and to increase the sample size within a class. S3.3 contains all the plots produced from this work, including three from TCGA and three from METABRIC. The blue solid lines in the dendrograms show preferred merges by BHC (clusters). Red dashed line show merges further up the cluster hierarchy. The numbers on the branches are the log odds for merging. S3.4 contains the clustering results from the three TCGA analyses and an .pdf image illustrating a Venn diagram with the overlaps between the different TCGA Basal dominant classes. Each pairwise analysis is organised into its analysis folder, containing a Data folder (for the input files) and a Working_directory folder (for the output files). The Data folder contains a relevant median-centred patient expression data (.csv file). The Working_directory folder contains two text files (.txt) describing the members of patients or genes in the resulting clusters, two resulting plots (.pdf files) and R data (.RDa files) produced while running the code (provided in S3.6). Each analysis folder also contains a patients_hc.pdf file that illustrates the hierarchical structure for the patient clusters. This was generated separately for visualisation purposes using the plot() function and hc_b.Rda is in the Working_directory as the data. S3.5 contains clustering results from the three METABRIC analyses. The folder organisations and files contained in this supplementary are equivalent to S3.4 above. S3.6 contains the Clustering_code_using_BHC.R with written descriptions and remarks in code, provided for reproducible research. (ZIP) [file pone.0252901.s003.zip › S3.1_Cluster_analysis_strategies.zip!/S_3_1_Cluster_analysis_strategies/S_3_1_Cluster_analysis_strategies_Figure & Text.pdf]

Non-parametric multinomial Bayesian Hierarchical Clustering of patient-gene expression data can be performed in two-dimensions giving rise to two Cluster Analysis Strategies (where, n x p is a square). These two strategies, based on two different marginal likelihood estimations, result in two distinct discretised plots with lower and upper bounds, with the resulting dendrograms on alternative axes (figure on the next page). One clustering strategy uses the *transform* of the dataset to the other. In one analysis, patients are clustered according to the information content in the gene expression distribution profiles (parametric distributions, based on z-scores). The complementary analysis results in clustering genes according to information content from the (non-parametric distributions) patient profiles. These Cluster analyses are different.

The focus of our paper has been the first clustering strategy, to stratify the (n) patients according to the (p) NR gene expressions to reveal heterogeneous transcriptional patterns in different BC subtypes that are then used to identify critical NR functional network motifs, solely implicated in Basal-like patients. Our approach uses a pair-wise partial correlation between the NR gene expressions to reveal the NR associations as critical network motifs. Our highlighted networks are not only enriched in Basal-specific networks but are the most critical NR interactions for drug target discovery. These identified motifs do not provide indications of, nor prediction measures for, patients' clinical outcome.

The second clustering strategy is complementary and uses the *transform* of the dataset, to cluster the (n) NR genes according to the (p) patient profiles. This approach would cluster the genes into distinct expression patterns. Associations of patients could be found using a non-parametric equivalent of the pair-wise correlation (analogous to the above) and furthermore could relate to survival and the patients' clinical outcome characteristics for the different BC subtypes. A cursory view of the discretised plot can illustrate gene clusters and the patients stratified in either extreme (lower and upper bounds) in the NR expression. From the example shown in the figure on the next page, Gene Cluster 1 is highly expressed in Luminal A patients and are lowly expressed in Basal-like subtype. The distinctive expression behaviour between BC subtypes can provide a useful guide into potential biomarker determination. The clustering resulting from BHC opens up a novel avenue of transcriptome research and offers opportunities in a range of fields.

Our current work and Cluster Analysis Strategy 1

Alternative Cluster Analysis Strategy 2

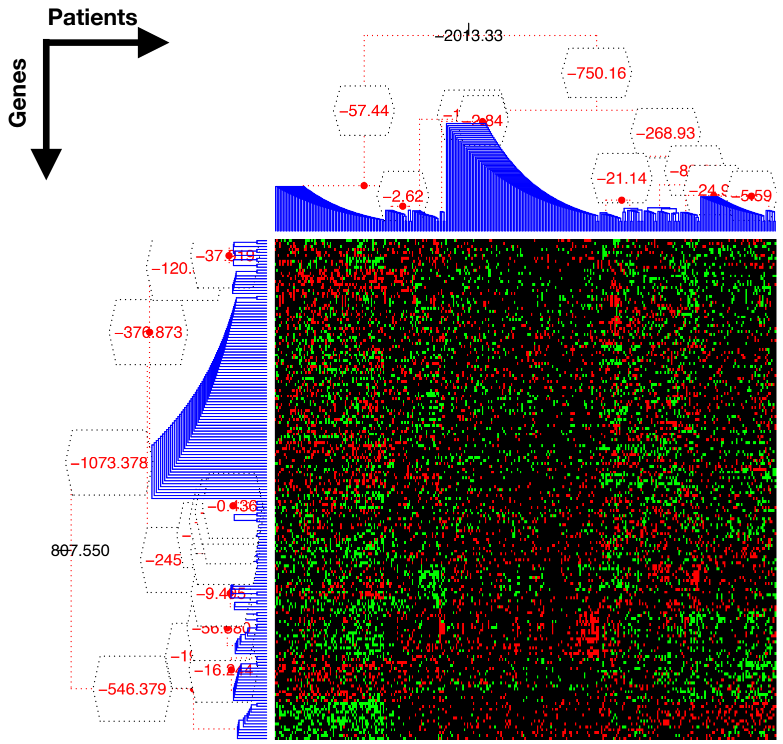

Patients clustered by NR genes

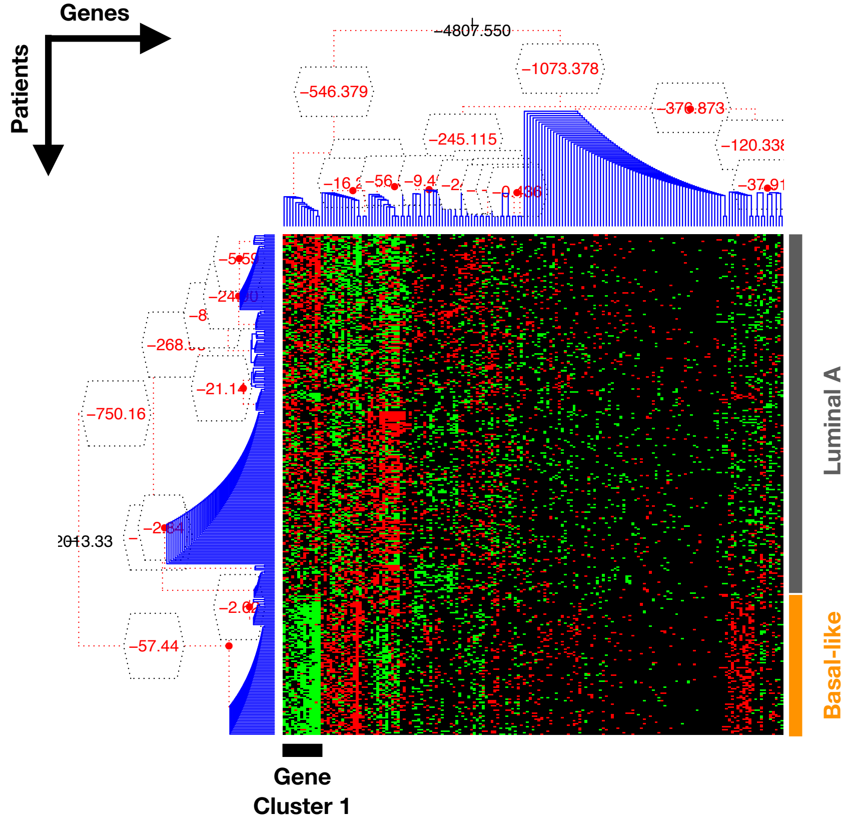

NR genes clustered by patients
