# Supplementary material for: Revealing nuclear receptor hub modules from Basal-like breast cancer expression networks
Source: PLoS One. 2021 Jun 23;16(6):e0252901. doi: 10.1371/journal.pone.0252901 (PMC8221501; doi:10.1371/journal.pone.0252901)
Supplement: S3 Appendix — This contains six sub-folders, including: S3.1 explains the two-dimensional clustering analysis that can be performed using the patient-expression data. S3.2 describes the process of consolidating clusters to classes. This was performed to reduce the number of clusters and to increase the sample size within a class. S3.3 contains all the plots produced from this work, including three from TCGA and three from METABRIC. The blue solid lines in the dendrograms show preferred merges by BHC (clusters). Red dashed line show merges further up the cluster hierarchy. The numbers on the branches are the log odds for merging. S3.4 contains the clustering results from the three TCGA analyses and an .pdf image illustrating a Venn diagram with the overlaps between the different TCGA Basal dominant classes. Each pairwise analysis is organised into its analysis folder, containing a Data folder (for the input files) and a Working_directory folder (for the output files). The Data folder contains a relevant median-centred patient expression data (.csv file). The Working_directory folder contains two text files (.txt) describing the members of patients or genes in the resulting clusters, two resulting plots (.pdf files) and R data (.RDa files) produced while running the code (provided in S3.6). Each analysis folder also contains a patients_hc.pdf file that illustrates the hierarchical structure for the patient clusters. This was generated separately for visualisation purposes using the plot() function and hc_b.Rda is in the Working_directory as the data. S3.5 contains clustering results from the three METABRIC analyses. The folder organisations and files contained in this supplementary are equivalent to S3.4 above. S3.6 contains the Clustering_code_using_BHC.R with written descriptions and remarks in code, provided for reproducible research. (ZIP) [file pone.0252901.s003.zip › S3.3_BHC_plots.zip!/S_3_3_BHC_plots/S_3_3_BHC_plots_Figure.pdf]

TCGA

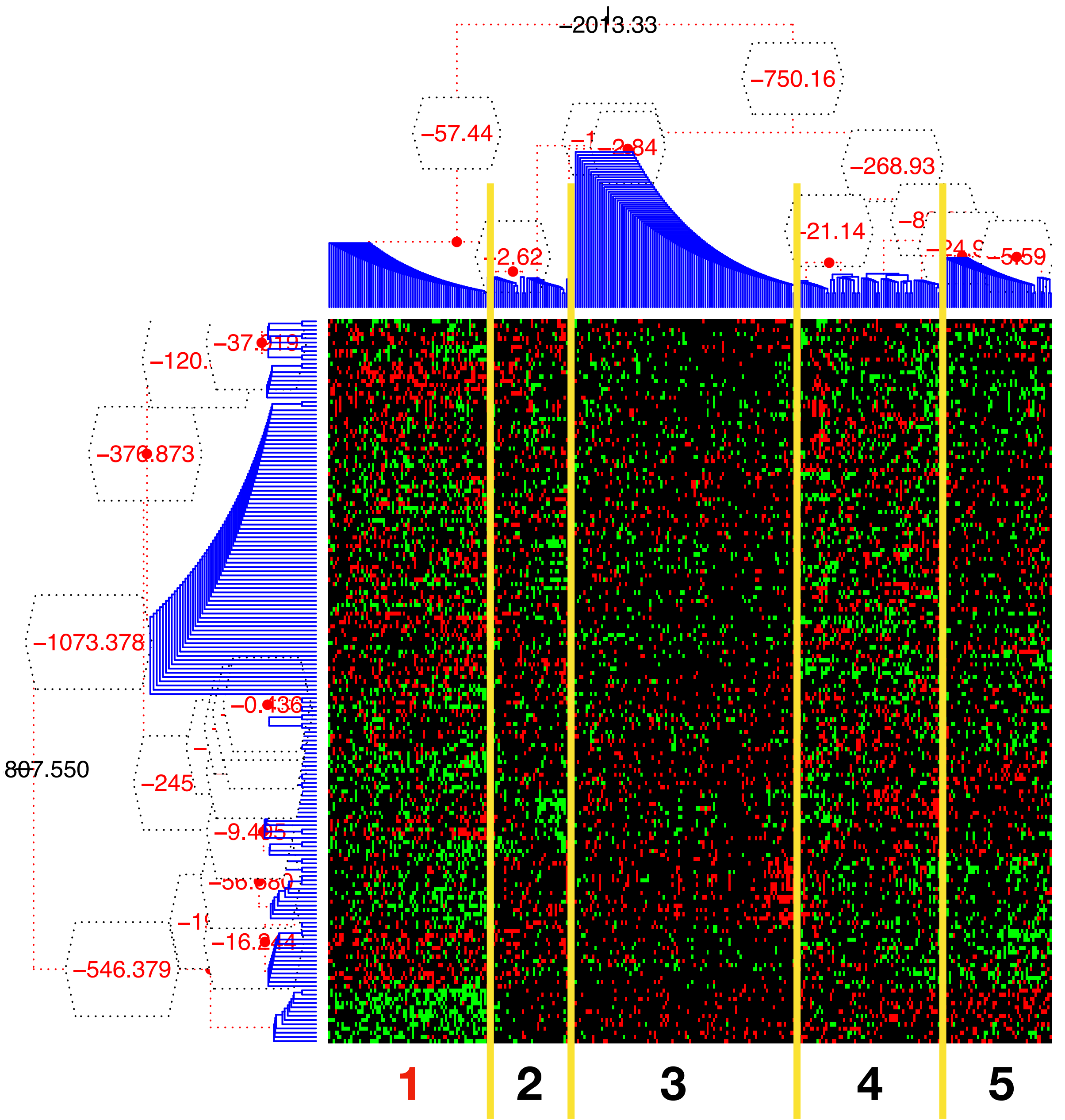

METABRIC

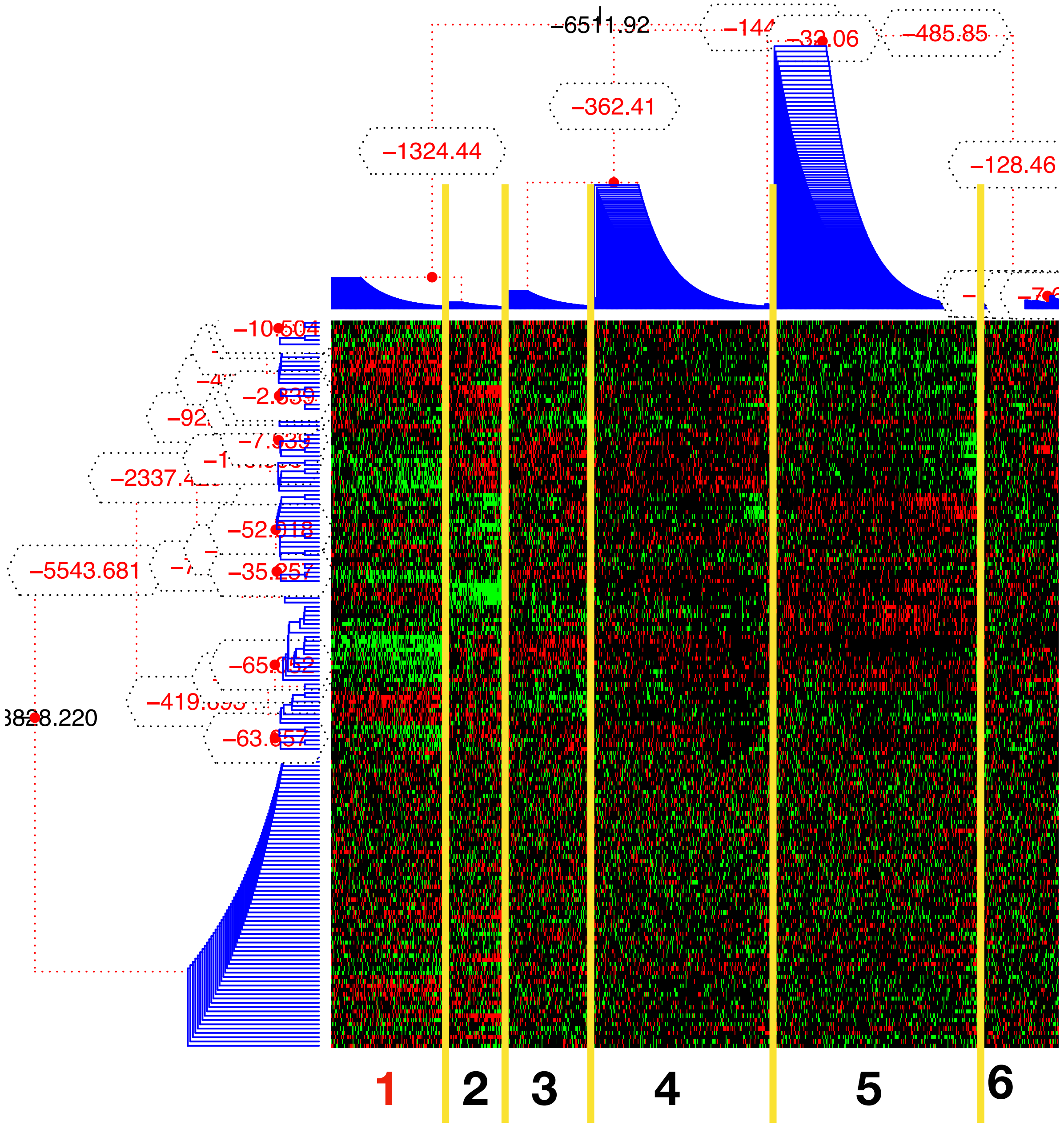

classes	1	2	3	4	5
Basal	89	3	1	5	0
Luminal A	1	15	104	24	87

classes	1	2	3	4	5	6
Basal	139	23	0	1	11	25
Luminal A	0	48	106	206	247	72

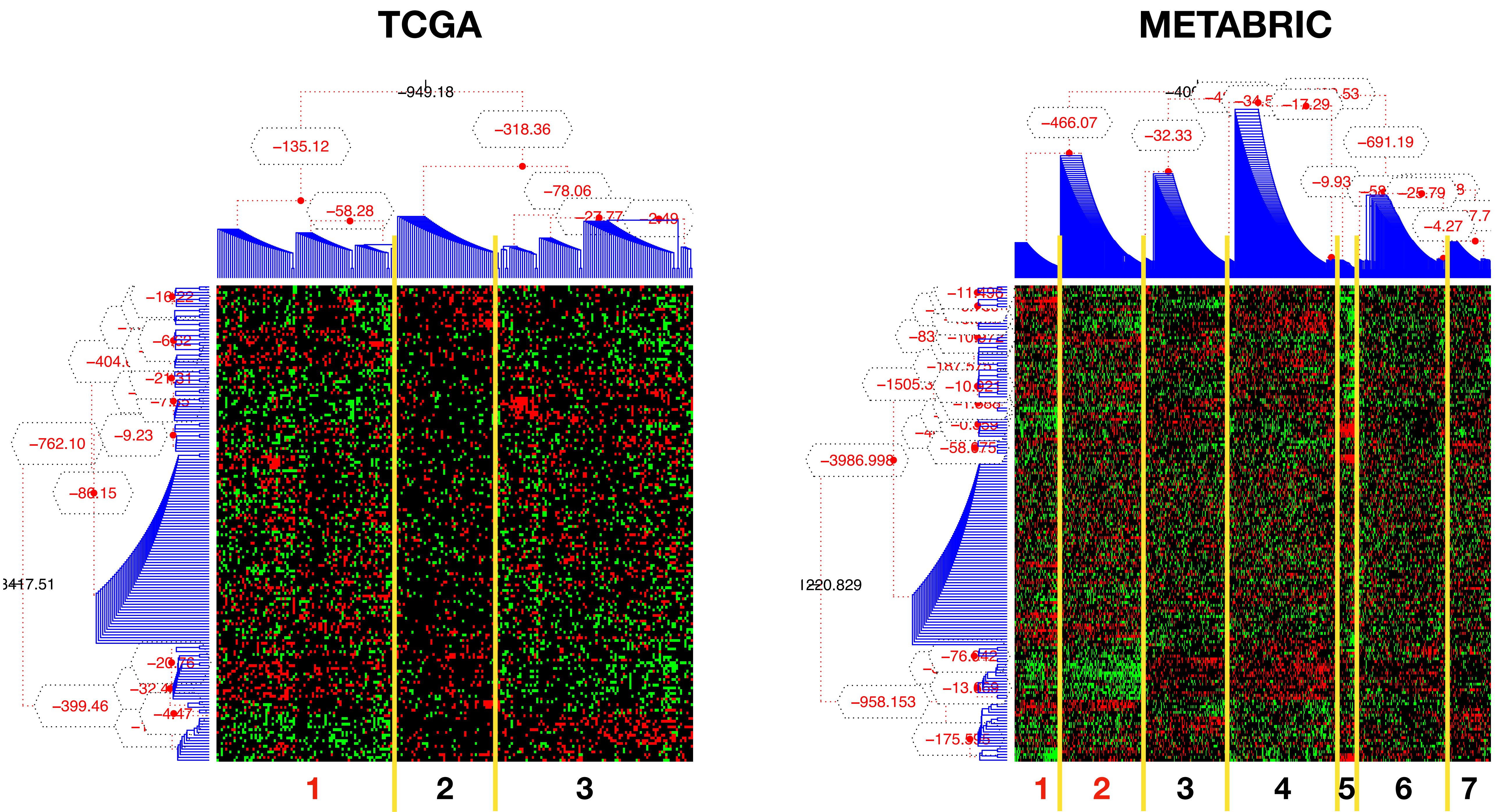

classes	1	2	3
Basal	80	17	1
Luminal B	5	32	90

classes	1	2	3	4	5	6	7
Basal	50	111	0	1	2	7	28
Luminal B	13	3	116	153	26	120	30

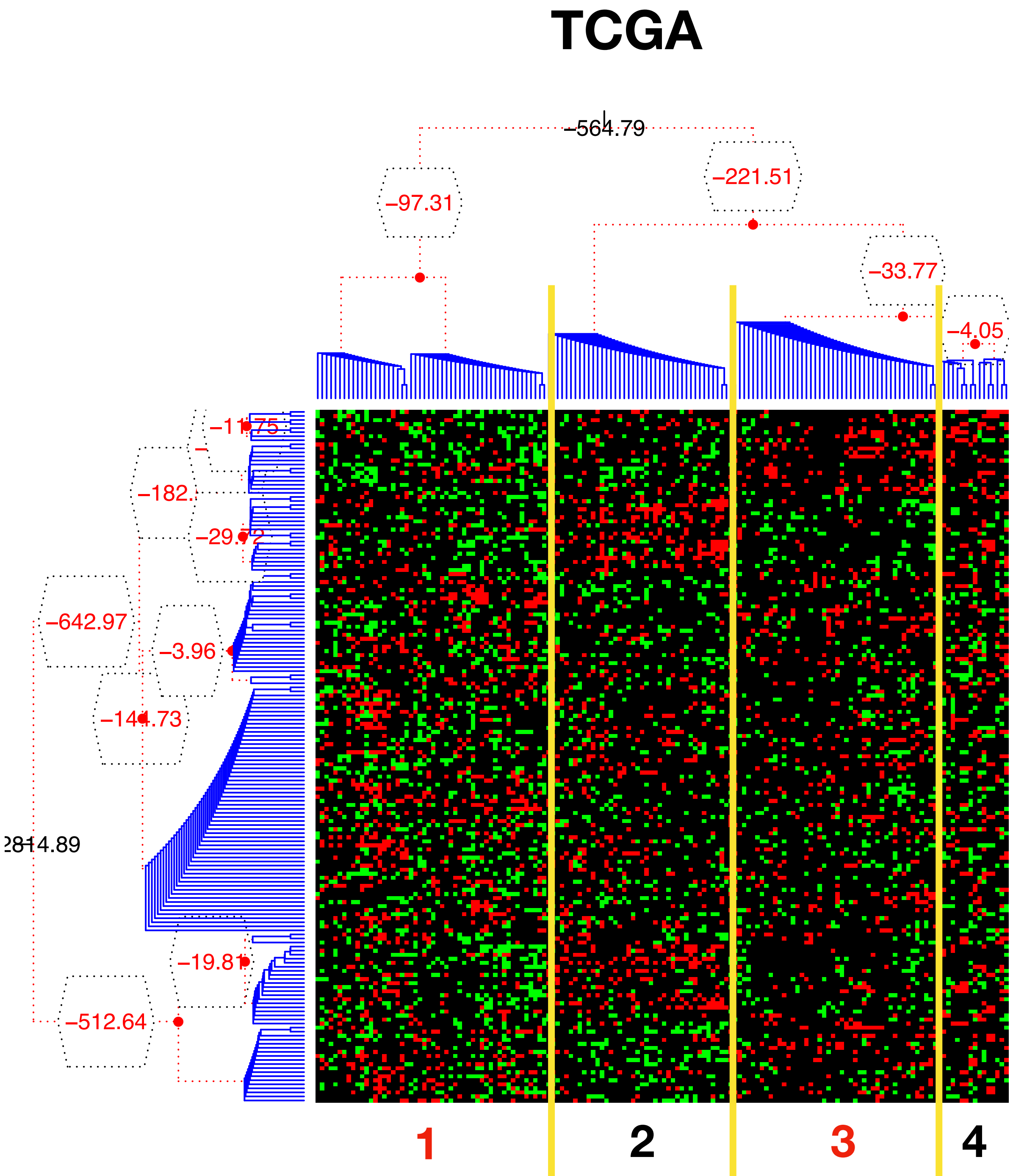

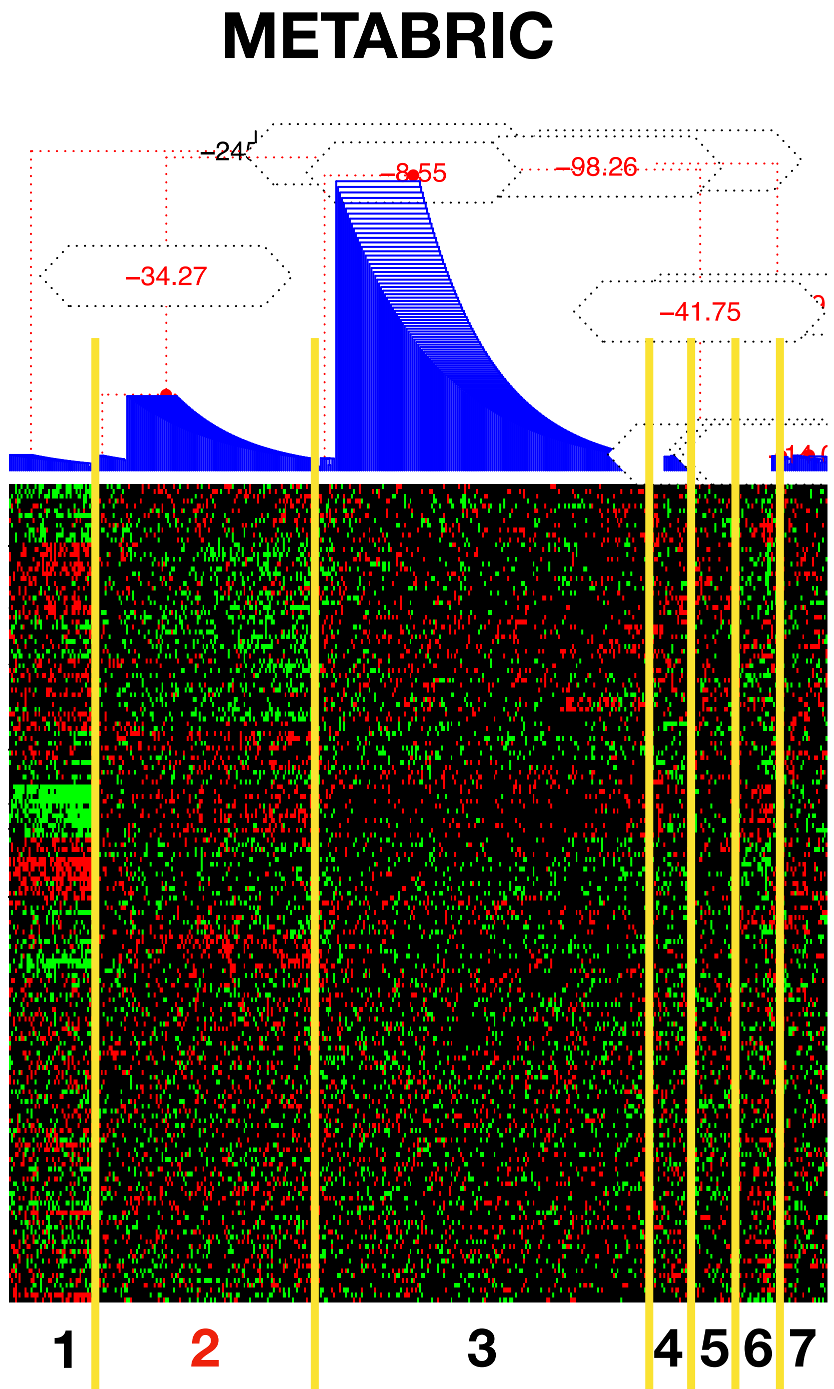

classes	1	2	3	4
Basal	45	1	43	9
Her2	7	40	4	7

classes	1	2	3	4	5	6	7
Basal	11	115	46	3	4	7	13
Her2	32	1	130	15	17	9	16
