# Supplementary material for: Revealing nuclear receptor hub modules from Basal-like breast cancer expression networks
Source: PLoS One. 2021 Jun 23;16(6):e0252901. doi: 10.1371/journal.pone.0252901 (PMC8221501; doi:10.1371/journal.pone.0252901)
Supplement: S3 Appendix — This contains six sub-folders, including: S3.1 explains the two-dimensional clustering analysis that can be performed using the patient-expression data. S3.2 describes the process of consolidating clusters to classes. This was performed to reduce the number of clusters and to increase the sample size within a class. S3.3 contains all the plots produced from this work, including three from TCGA and three from METABRIC. The blue solid lines in the dendrograms show preferred merges by BHC (clusters). Red dashed line show merges further up the cluster hierarchy. The numbers on the branches are the log odds for merging. S3.4 contains the clustering results from the three TCGA analyses and an .pdf image illustrating a Venn diagram with the overlaps between the different TCGA Basal dominant classes. Each pairwise analysis is organised into its analysis folder, containing a Data folder (for the input files) and a Working_directory folder (for the output files). The Data folder contains a relevant median-centred patient expression data (.csv file). The Working_directory folder contains two text files (.txt) describing the members of patients or genes in the resulting clusters, two resulting plots (.pdf files) and R data (.RDa files) produced while running the code (provided in S3.6). Each analysis folder also contains a patients_hc.pdf file that illustrates the hierarchical structure for the patient clusters. This was generated separately for visualisation purposes using the plot() function and hc_b.Rda is in the Working_directory as the data. S3.5 contains clustering results from the three METABRIC analyses. The folder organisations and files contained in this supplementary are equivalent to S3.4 above. S3.6 contains the Clustering_code_using_BHC.R with written descriptions and remarks in code, provided for reproducible research. (ZIP) [file pone.0252901.s003.zip › S3.5_METABRIC.zip!/S_3_5_METABRIC/Basal_vs_LuminalA/Working_directory/Rplot_169 Genes by 878 Patients (0.085, 0.83, 0.085)_a.pdf]

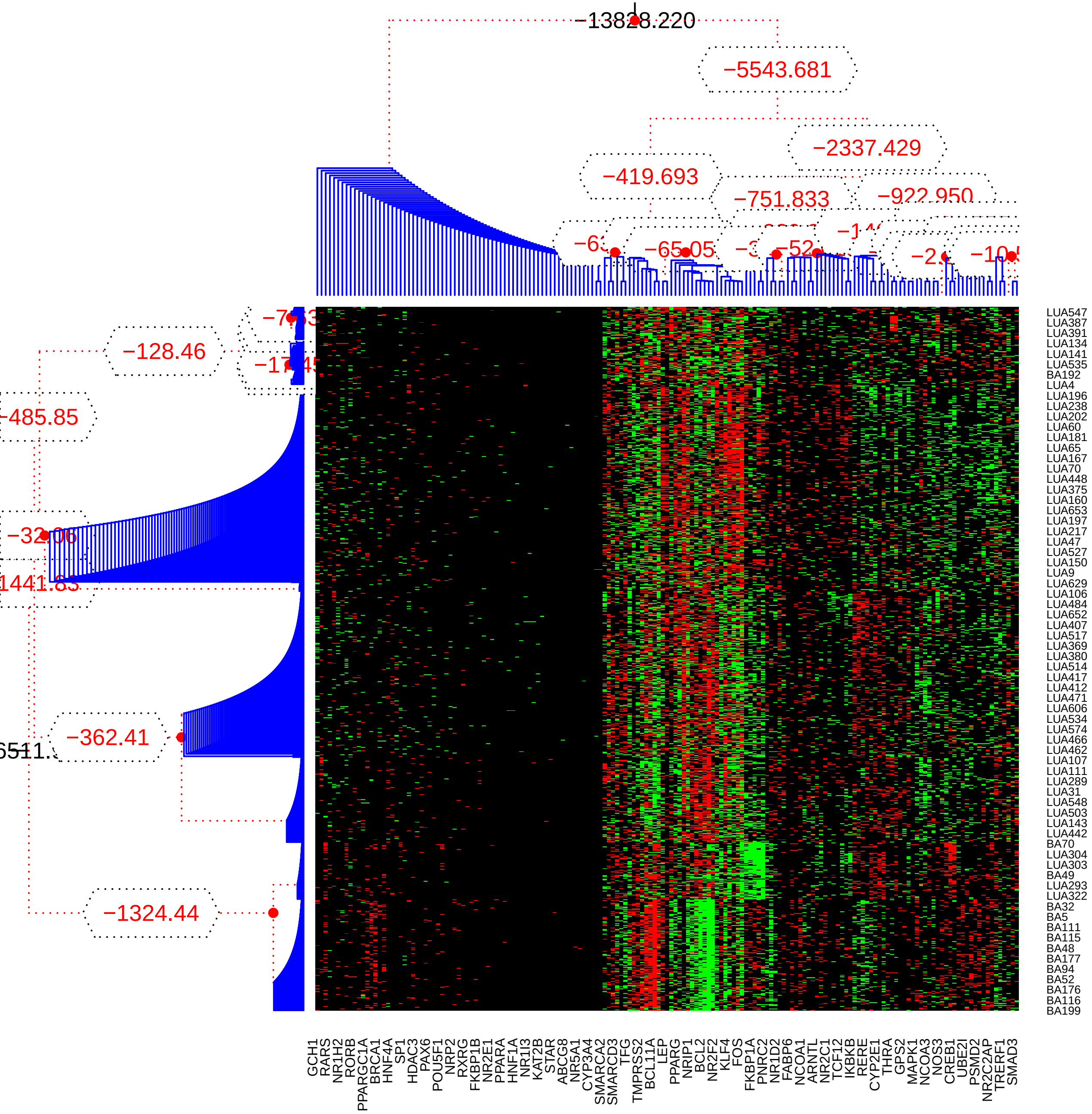
−13828.220
−5543.681
−2337.429
−419.693
−751.833
−922.950
−128.46
−485.85
−362.41
−1324.44
LUA547
LUA387
LUA391
LUA134
LUA141
LUA535
BA192
LUA4
LUA196
LUA238
LUA202
LUA60
LUA181
LUA65
LUA167
LUA70
LUA448
LUA375
LUA160
LUA653
LUA197
LUA217
LUA47
LUA527
LUA150
LUA9
LUA629
LUA106
LUA484
LUA652
LUA407
LUA517
LUA369
LUA380
LUA514
LUA417
LUA412
LUA471
LUA606
LUA534
LUA574
LUA466
LUA462
LUA107
LUA111
LUA289
LUA31
LUA548
LUA503
LUA143
LUA442
BA70
LUA304
LUA303
BA49
LUA293
LUA322
BA32
BA5
BA111
BA115
BA48
BA177
BA94
BA52
BA176
BA116
BA199
GCH1
RARS
NR1H2
RORB
PPARGC1A
BRCA1
HNF4A
SP1
HDAC3
PAX6
POU5F1
NRP2
RXRG
FKBP1B
NR2E1
PPARA
HNF1A
NR1I3
KAT2B
STAR
ABCG8
NR5A1
CYP3A4
SMARCA2
SMARCD3
TFG
TMPRSS2
BCL11A
LEP
PPARG
NRIP1
BCL2
NR2F2
KLF4
FOS
FKBP1A
PNRC2
NR1D2
FABP6
NCOA1
ARNTL
NR2C1
TCF12
IKBKB
RERE
CYP2E1
THRA
GPS2
MAPK1
NCOA3
NOS3
CREB1
UBE2I
PSMD2
NR2C2AP
TRERF1
SMAD3
