# Supplementary material for: Revealing nuclear receptor hub modules from Basal-like breast cancer expression networks
Source: PLoS One. 2021 Jun 23;16(6):e0252901. doi: 10.1371/journal.pone.0252901 (PMC8221501; doi:10.1371/journal.pone.0252901)
Supplement: S3 Appendix — This contains six sub-folders, including: S3.1 explains the two-dimensional clustering analysis that can be performed using the patient-expression data. S3.2 describes the process of consolidating clusters to classes. This was performed to reduce the number of clusters and to increase the sample size within a class. S3.3 contains all the plots produced from this work, including three from TCGA and three from METABRIC. The blue solid lines in the dendrograms show preferred merges by BHC (clusters). Red dashed line show merges further up the cluster hierarchy. The numbers on the branches are the log odds for merging. S3.4 contains the clustering results from the three TCGA analyses and an .pdf image illustrating a Venn diagram with the overlaps between the different TCGA Basal dominant classes. Each pairwise analysis is organised into its analysis folder, containing a Data folder (for the input files) and a Working_directory folder (for the output files). The Data folder contains a relevant median-centred patient expression data (.csv file). The Working_directory folder contains two text files (.txt) describing the members of patients or genes in the resulting clusters, two resulting plots (.pdf files) and R data (.RDa files) produced while running the code (provided in S3.6). Each analysis folder also contains a patients_hc.pdf file that illustrates the hierarchical structure for the patient clusters. This was generated separately for visualisation purposes using the plot() function and hc_b.Rda is in the Working_directory as the data. S3.5 contains clustering results from the three METABRIC analyses. The folder organisations and files contained in this supplementary are equivalent to S3.4 above. S3.6 contains the Clustering_code_using_BHC.R with written descriptions and remarks in code, provided for reproducible research. (ZIP) [file pone.0252901.s003.zip › S3.5_METABRIC.zip!/S_3_5_METABRIC/Basal_vs_LuminalA/Working_directory/Rplot_878 Patients by 169 Genes (0.13, 0.74, 0.13)_b.pdf]

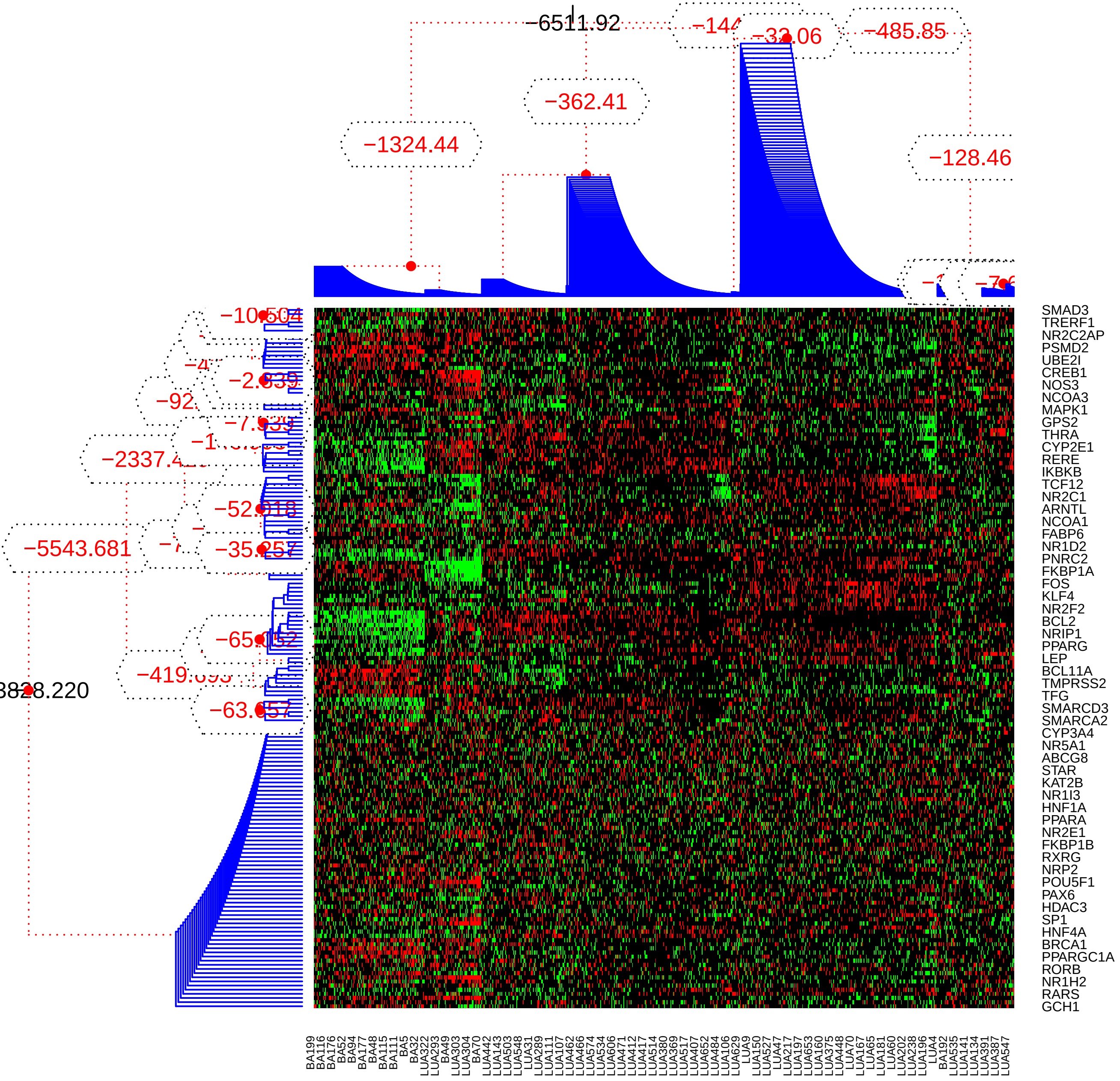
−6511.92
−1324.44
−362.41
−485.85
−128.46
−5543.681
SMAD3
TRERF1
NR2C2AP
PSMD2
UBE2I
CREB1
NOS3
NCOA3
MAPK1
GPS2
THRA
CYP2E1
RERE
IKBKB
TCF12
NR2C1
ARNTL
NCOA1
FABP6
NR1D2
PNRC2
FKBP1A
FOS
KLF4
NR2F2
BCL2
NRIP1
PPARG
LEP
BCL11A
TMPRSS2
TFG
SMARCD3
SMARCA2
CYP3A4
NR5A1
ABCG8
STAR
KAT2B
NR1I3
HNF1A
PPARA
NR2E1
FKBP1B
RXRG
NRP2
POU5F1
PAX6
HDAC3
SP1
HNF4A
BRCA1
PPARGC1A
RORB
NR1H2
RARS
GCH1
BA199
BA116
BA176
BA52
BA94
BA177
BA48
BA115
BA111
BA5
BA32
LUA322
LUA293
BA49
LUA303
LUA304
BA70
LUA442
LUA143
LUA503
LUA548
LUA31
LUA289
LUA111
LUA107
LUA462
LUA466
LUA574
LUA534
LUA606
LUA471
LUA412
LUA417
LUA514
LUA380
LUA369
LUA517
LUA407
LUA652
LUA484
LUA106
LUA629
LUA9
LUA150
LUA527
LUA47
LUA217
LUA197
LUA653
LUA160
LUA375
LUA448
LUA70
LUA167
LUA65
LUA181
LUA60
LUA202
LUA238
LUA196
LUA4
BA192
LUA535
LUA141
LUA134
LUA391
LUA387
LUA547
