# Supplementary material for: Revealing nuclear receptor hub modules from Basal-like breast cancer expression networks
Source: PLoS One. 2021 Jun 23;16(6):e0252901. doi: 10.1371/journal.pone.0252901 (PMC8221501; doi:10.1371/journal.pone.0252901)
Supplement: S3 Appendix — This contains six sub-folders, including: S3.1 explains the two-dimensional clustering analysis that can be performed using the patient-expression data. S3.2 describes the process of consolidating clusters to classes. This was performed to reduce the number of clusters and to increase the sample size within a class. S3.3 contains all the plots produced from this work, including three from TCGA and three from METABRIC. The blue solid lines in the dendrograms show preferred merges by BHC (clusters). Red dashed line show merges further up the cluster hierarchy. The numbers on the branches are the log odds for merging. S3.4 contains the clustering results from the three TCGA analyses and an .pdf image illustrating a Venn diagram with the overlaps between the different TCGA Basal dominant classes. Each pairwise analysis is organised into its analysis folder, containing a Data folder (for the input files) and a Working_directory folder (for the output files). The Data folder contains a relevant median-centred patient expression data (.csv file). The Working_directory folder contains two text files (.txt) describing the members of patients or genes in the resulting clusters, two resulting plots (.pdf files) and R data (.RDa files) produced while running the code (provided in S3.6). Each analysis folder also contains a patients_hc.pdf file that illustrates the hierarchical structure for the patient clusters. This was generated separately for visualisation purposes using the plot() function and hc_b.Rda is in the Working_directory as the data. S3.5 contains clustering results from the three METABRIC analyses. The folder organisations and files contained in this supplementary are equivalent to S3.4 above. S3.6 contains the Clustering_code_using_BHC.R with written descriptions and remarks in code, provided for reproducible research. (ZIP) [file pone.0252901.s003.zip › S3.5_METABRIC.zip!/S_3_5_METABRIC/Basal_vs_LuminalB/Working_Directory/Rplot_660 Patients by 169 Genes (0.135, 0.73, 0.135)_b.pdf]

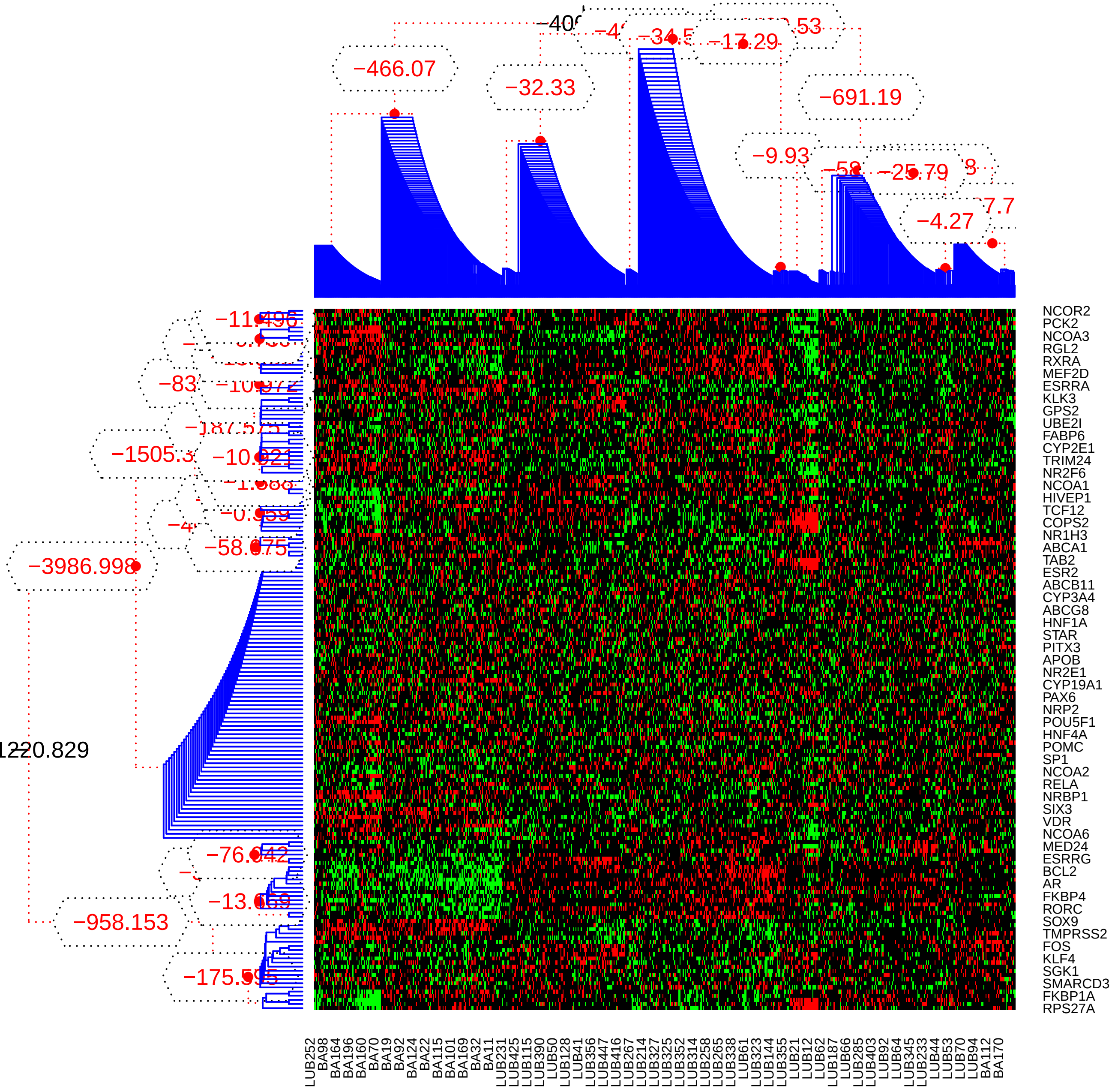

−466.07
−32.33
−691.19
−9.93
−25.79
−4.27
−3986.998
−958.153
NCOR2
PCK2
NCOA3
RGL2
RXRA
MEF2D
ESRRA
KLK3
GPS2
UBE2I
FABP6
CYP2E1
TRIM24
NR2F6
NCOA1
HIVEP1
TCF12
COPS2
NR1H3
ABCA1
TAB2
ESR2
ABCB11
CYP3A4
ABCG8
HNF1A
STAR
PITX3
APOB
NR2E1
CYP19A1
PAX6
NRP2
POU5F1
HNF4A
POMC
SP1
NCOA2
RELA
NRBP1
SIX3
VDR
NCOA6
MED24
ESRRG
BCL2
AR
FKBP4
RORC
SOX9
TMPRSS2
FOS
KLF4
SGK1
SMARCD3
FKBP1A
RPS27A
LUB252
BA98
BA164
BA196
BA160
BA70
BA19
BA92
BA124
BA22
BA115
BA101
BA169
BA32
BA11
LUB231
LUB425
LUB115
LUB390
LUB50
LUB128
LUB41
LUB356
LUB447
LUB416
LUB267
LUB214
LUB327
LUB325
LUB352
LUB314
LUB258
LUB265
LUB338
LUB61
LUB323
LUB144
LUB355
LUB21
LUB12
LUB62
LUB187
LUB66
LUB285
LUB403
LUB92
LUB64
LUB345
LUB233
LUB44
LUB53
LUB70
LUB94
BA112
BA170
