# Supplementary material for: Revealing nuclear receptor hub modules from Basal-like breast cancer expression networks
Source: PLoS One. 2021 Jun 23;16(6):e0252901. doi: 10.1371/journal.pone.0252901 (PMC8221501; doi:10.1371/journal.pone.0252901)
Supplement: S3 Appendix — This contains six sub-folders, including: S3.1 explains the two-dimensional clustering analysis that can be performed using the patient-expression data. S3.2 describes the process of consolidating clusters to classes. This was performed to reduce the number of clusters and to increase the sample size within a class. S3.3 contains all the plots produced from this work, including three from TCGA and three from METABRIC. The blue solid lines in the dendrograms show preferred merges by BHC (clusters). Red dashed line show merges further up the cluster hierarchy. The numbers on the branches are the log odds for merging. S3.4 contains the clustering results from the three TCGA analyses and an .pdf image illustrating a Venn diagram with the overlaps between the different TCGA Basal dominant classes. Each pairwise analysis is organised into its analysis folder, containing a Data folder (for the input files) and a Working_directory folder (for the output files). The Data folder contains a relevant median-centred patient expression data (.csv file). The Working_directory folder contains two text files (.txt) describing the members of patients or genes in the resulting clusters, two resulting plots (.pdf files) and R data (.RDa files) produced while running the code (provided in S3.6). Each analysis folder also contains a patients_hc.pdf file that illustrates the hierarchical structure for the patient clusters. This was generated separately for visualisation purposes using the plot() function and hc_b.Rda is in the Working_directory as the data. S3.5 contains clustering results from the three METABRIC analyses. The folder organisations and files contained in this supplementary are equivalent to S3.4 above. S3.6 contains the Clustering_code_using_BHC.R with written descriptions and remarks in code, provided for reproducible research. (ZIP) [file pone.0252901.s003.zip › S3.5_METABRIC.zip!/S_3_5_METABRIC/Basal_vs_LuminalB/Working_Directory/Rplot_169 Genes by 660 Patients (0.09, 0.82, 0.09)_a.pdf]

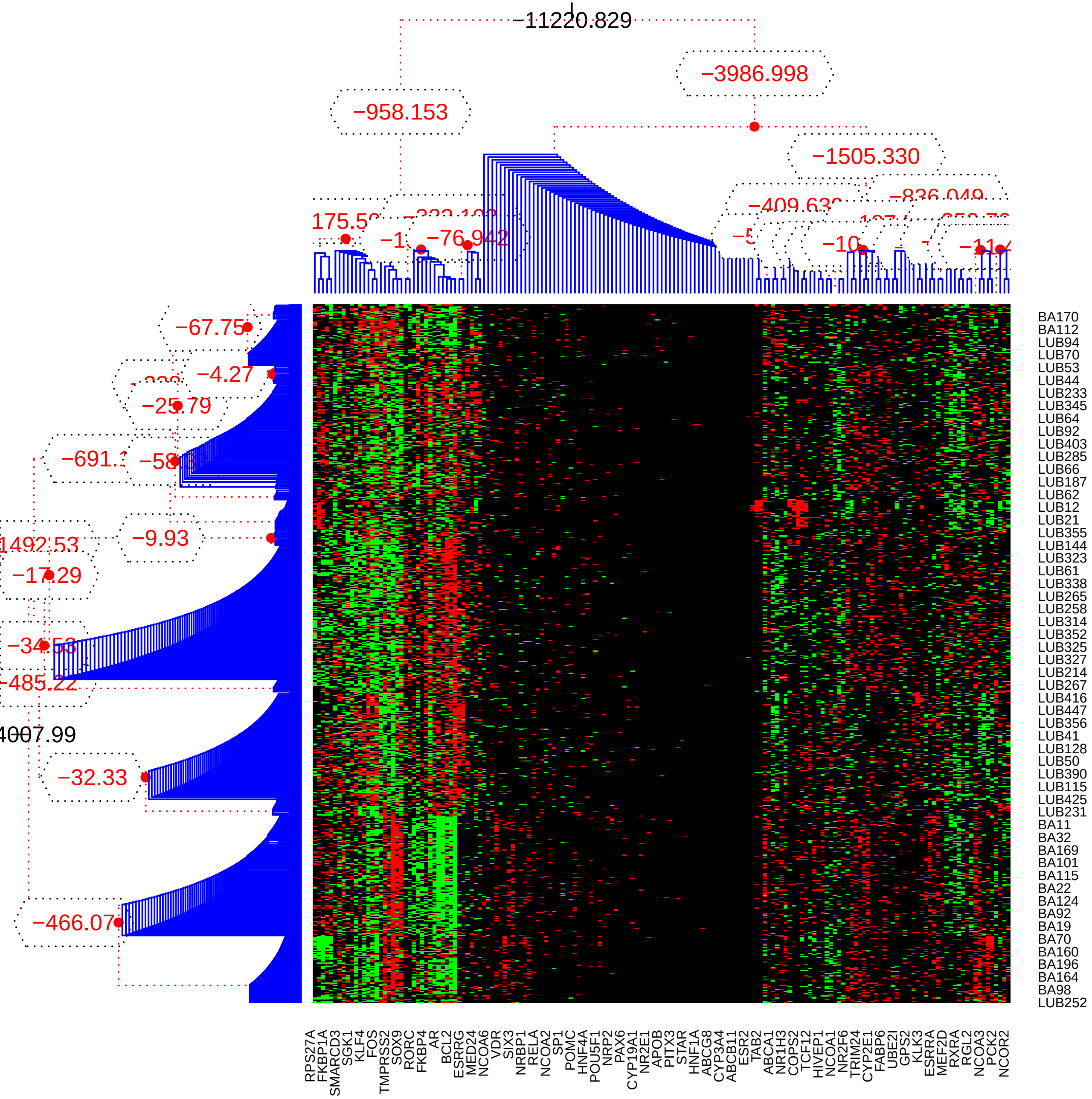

−11220.829
−3986.998
−958.153
−1505.330
−76.942
−67.75
−4.27
−25.79
−9.93
−17.29
−485.22
4007.99
−32.33
−466.07
BA170
BA112
LUB94
LUB70
LUB53
LUB44
LUB233
LUB345
LUB64
LUB92
LUB403
LUB285
LUB66
LUB187
LUB62
LUB12
LUB21
LUB355
LUB144
LUB323
LUB61
LUB338
LUB265
LUB258
LUB314
LUB352
LUB325
LUB327
LUB214
LUB267
LUB416
LUB447
LUB356
LUB41
LUB128
LUB50
LUB390
LUB115
LUB425
LUB231
BA11
BA32
BA169
BA101
BA115
BA22
BA124
BA92
BA19
BA70
BA160
BA196
BA164
BA98
LUB252
RPS27A
FKBP1A
SMARCD3
SGK1
KLF4
FOS
TMPRSS2
SOX9
RORC
FKBP4
AR
BCL2
ESRRG
MED24
NCOA6
VDR
SIX3
NRBP1
RELA
NCOA2
SP1
POMC
HNF4A
POU5F1
NRP2
PAX6
CYP19A1
NR2E1
APOB
PITX3
STAR
HNF1A
ABCG8
CYP3A4
ABCB11
ESR2
TAB2
ABCA1
NR1H3
COPS2
TCF12
HIVEP1
NCOA1
NR2F6
TRIM24
CYP2E1
FABP6
UBE2I
GPS2
KLK3
ESRRA
MEF2D
RXRA
RGL2
NCOA3
PCK2
NCOR2
